# Preclinical pharmacology of alogabat: a novel GABA_A_-α5 positive allosteric modulator targeting neurodevelopmental disorders with impaired GABA_A_ signaling

**DOI:** 10.3389/fphar.2025.1626078

**Published:** 2025-07-21

**Authors:** Giuseppe Cecere, Theresa M. Ballard, Frederic Knoflach, Michael Honer, Joerg F. Hipp, Pilar Garces, Thomas Mueggler, Basil Künnecke, Andreas Bruns, Eric P. Prinssen, Philipp Schoenenberger, Philipp Janz, Roger Redondo, Barbara Biemans, Henner Knust, Andrés Olivares-Morales, Alessandro Brigo, Jan Michael Schulz, Daniel Bertrand, Michael Saxe, Eoin C. O’Connor, Maria-Clemencia Hernandez

**Affiliations:** ^1^ Medicinal Chemistry, Roche Pharmaceutical Research and Early Development, Roche Innovation Center Basel, Basel, Switzerland; ^2^ Neuroscience and Rare Diseases, Roche Pharmaceutical Research and Early Development, Roche Innovation Center Basel, Basel, Switzerland; ^3^ Pharmaceutical Sciences, Roche Pharmaceutical Research and Early Development, Roche Innovation Center Basel, Basel, Switzerland; ^4^ HiQScreen Sàrl, Vésenaz, Switzerland

**Keywords:** autism spectrum disorder, Angelman syndrome, GABA_A_, GABA_A_-α5, positron emission tomography (PET), electroencephalography, pharmacological MRI, biomarker

## Abstract

**Background:**

Alterations in the GABAergic system contribute to the pathophysiology of neurodevelopmental disorders, including autism spectrum disorder (ASD) and Angelman syndrome (AS), particularly in cases involving large deletions in the 15q11–13 region. Positive modulation of GABA_A_-α5 receptors may provide a novel therapeutic approach without the typical side effects associated with non-selective GABA_A_ positive allosteric modulators such as diazepam.

**Methods:**

Alogabat was assessed for binding and functional activity at GABA_A_-α5β3γ2 receptors *in vitro* and in electrophysiological studies using hippocampal slices. *In vivo* studies in rodents included receptor occupancy (RO) using a selective GABA_A_-α5 tracer (autoradiography), pharmacological MRI, and electroencephalography (EEG). Alogabat was evaluated for its effects on the repetitive behavior phenotype in BTBR and contactin-associated protein-like 2 (Cntnap2^−/−^) knockout mice, seizure models, cognitive performance in rats, and rotarod performance following combination treatment with diazepam.

**Results:**

Alogabat is a potent positive allosteric modulator of GABA_A_-α5 receptors, with binding and functional selectivity. Receptor occupancy studies provided direct proof of dose-dependent target engagement. Functional circuit modulation was demonstrated by dose-dependent regional perfusion changes in pharmacological MRI and changes in EEG theta- and beta-band power in rats. At >50% GABA_A_-α5 receptor occupancy, alogabat normalized elevated self-grooming behavior in both Cntnap2^−/−^ and BTBR mice and exhibited antiepileptic activity in rats. Alogabat did not impair cognition in wildtype rats at GABA_A_-α5 receptor occupancy up to 75%, although impairment occurred at higher doses, probably due to increased activity at other receptor subtypes and/or saturation of α5 receptors. Alogabat did not worsen diazepam-induced impairment on the rotarod test.

**Conclusion:**

Alogabat showed beneficial effects in mouse models relevant to neurodevelopmental disorders and anti-seizure activity at doses that did not produce cognitive, sedative, or motoric side effects.

## 1 Introduction

Alterations in the GABAergic system, the main inhibitory neurotransmitter system in the brain, may contribute to the pathophysiology of neurodevelopmental disorders (NDDs), such as autism spectrum disorder (ASD) and Angelman syndrome (AS). ASD is a complex, heterogeneous NDD with both genetic and environmental factors (Bourgeron, 2015) contributing to aberrant changes in brain growth, neuronal development, and functional connectivity (Dhossche et al., 2002; Pizzarelli and Cherubini, 2011; Braat and Kooy, 2015; Robertson et al., 2016). ASD is characterized by two core domains: impairments in social interaction and communication and the presence of repetitive or restricted behaviors, interests, or activities (American Psychiatric Association, 2008). There are a range of comorbid conditions, including irritability, depression, anxiety, attention deficits, obsessive-compulsive symptoms, seizures, and sleep disruption. AS is a rare genetic NDD with a prevalence of 1 in 12,000 to 20,000 births (Luk and Lo, 2016; Mertz et al., 2013). Individuals with AS often present with autistic features, severe intellectual disability, microcephaly, speech impairment, bursts of laughter, sleep problems, movement disorders, and epilepsy (Buiting, 2010; Kalsner and Chamberlain, 2015).

GABA_A_ receptors are ligand-gated chloride channels. There are 19 genes encoding GABA_A_ receptor subunits that assemble as pentamers, with the most common stoichiometry being two α, two β, and one γ subunit. GABA_A_ subunit combinations give rise to functional, circuit, and behavioral specificity (Sieghart, 2006; Vithlani et al., 2011). GABA_A_ receptors that contain the α5 subunit (GABA_A_-α5) are of particular interest due to their restricted pattern of expression and unique physiological and pharmacological properties (Sur et al., 1999; Mohler, 2011). In humans, GABA_A_ receptors containing the α5 subunit are preferentially localized in the hippocampus, prefrontal cortex, nucleus accumbens, amygdala, insular cortex, anterior cingulate, and cingulate cortex—key regions believed to be involved in the neuropathology and pathophysiology of ASD (Mendez et al., 2013; Myers et al., 2017). The *GABRA5* gene has been proposed as a candidate gene for ASD (Delong, 2007; Mesbah-Oskui et al., 2017; Warrier et al., 2013; Di Nanni et al., 2019), suggesting decreased GABAergic inhibition in a subpopulation of autistic individuals. Mutations involving the GABA_A_ receptor subunit genes *GABRA5*, *GABRB3*, and *GABRG3* (encoding the α5, β3, and γ3 subunits) found on chromosomal region 15q11–q13 have been associated with NDDs including AS, epilepsy, and Prader–Willi syndrome in addition to ASD (Hogart et al., 2010; Sanders, 2015). AS is predominantly caused by the loss of function of the maternally inherited *UBE3A* gene; however, approximately 75% of individuals with AS carry a large deletion of the maternal 15q11–q13 chromosomal region. Clinical studies have shown that individuals with AS who have large deletions (deletion AS) exhibit more severe symptoms than those in whom *UBE3A* is the only gene affected, suggesting that the *GABRA5*, *GABRB3*, and *GABRG3* genes are relevant to the etiology of the disease.

Imaging studies using PET and magnetic resonance spectroscopy have shown reduced GABA_A_ receptor binding in the superior and medial frontal cortex (Mori et al., 2012) and reduced GABA levels in individuals with ASD (Robertson et al., 2016; Gaetz et al., 2014; Rojas et al., 2014; Puts et al., 2017). Postmortem studies have shown reduced numbers of inhibitory interneurons (Dufour et al., 2023), reduced expression of GABA_A_ receptor subunits (DeLorey, 2005; Abrahams and Geschwind, 2008), and reduced levels of the GABA-synthesizing enzymes such as glutamic acid decarboxylase (GAD) 65 and 67 (Fatemi et al., 2002). There is increasing evidence from genetic, environmental, and phenotypic rodent models relevant to ASD indicating that GABAergic circuit dysfunctions contribute to autism-like phenotypes (Fukuda et al., 2005; Chao et al., 2010; Banerjee et al., 2013). Such deficits may result from multiple causes, including a reduced number of GABAergic interneurons, altered interneuron function, impaired GABA_A_ receptor trafficking to the membrane, reduced expression of GABA_A_ receptor subunits, and reduced GABA uptake function of GAT-1 in neurons (Nakamura et al., 2016; Orefice et al., 2016; Mermer et al., 2021). Supportive evidence is provided by the observation of autism-like behaviors in both GABA_A_-α5 and β3 subunit knockout mice (Zurek et al., 2016; DeLorey et al., 2008). Regarding possible therapeutic approaches, enhancement of the GABA_A_ receptor activity by non-selective benzodiazepines has been shown to ameliorate autism-like behavioral deficits in mouse models; however, due to sedation that is likely mediated by the activation of the GABA_A_-α1 subtype, very narrow therapeutic margins were observed (Han et al., 2012; Han et al., 2014; Lim et al., 2017). Interestingly, a recent study showed that a GABA_A_-α5 positive allosteric modulator (PAM), SH-053-2′F-R-CH3, attenuated social and cognitive deficits in male rats exposed to valproic acid *in utero*, a rat model relevant to autism (Souza et al., 2024).

The aim of the current study was to determine whether a selective GABA_A_-α5 PAM, alogabat (also known as RO7017773 or RG7816), would produce beneficial effects in animal models relevant to autism-like repetitive behavior—specifically BTBR mice (McFarlane et al., 2008) and contactin-associated protein-like 2 [Cntnap2^−/−^] homozygous knockout mice (Penagarikano et al., 2011)—and anti-seizure activity, without the typical side effects of non-selective GABA_A_ PAMs, such as sedation and cognitive impairment. Furthermore, target engagement was assessed by *in vivo* receptor occupancy (RO) and pharmacological MRI (phMRI) in rodents, and an electroencephalography (EEG) study in rats was undertaken to identify a putative pharmacodynamic readout for future clinical trials.

## 2 Materials and methods

Further information for each method listed below can be found in the Supplementary Material.

### 2.1 Materials

**FIGURE 1:**
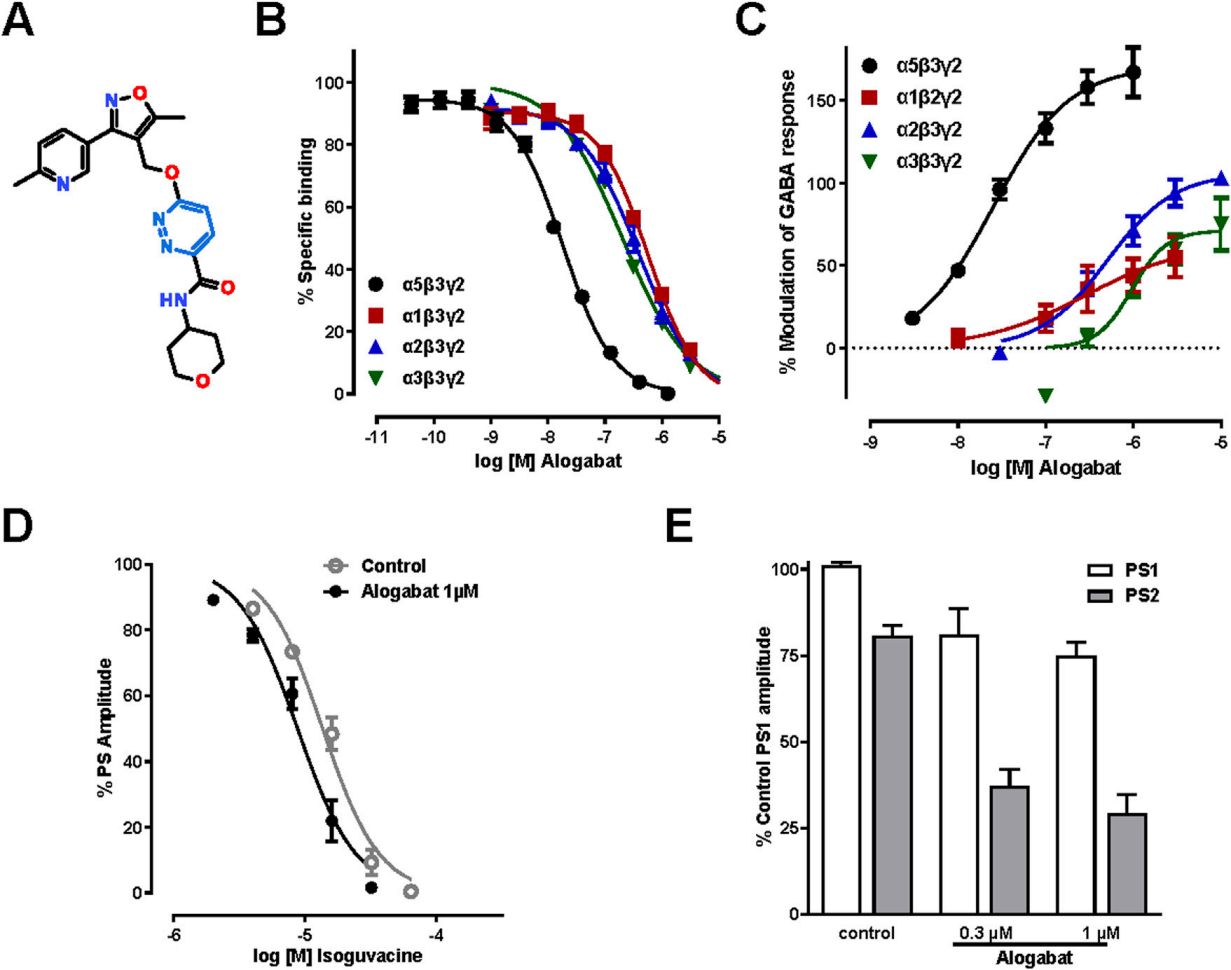
*In vitro* pharmacology of alogabat. **(A)** Chemical structure of alogabat (RO7017773; C21H23N5O4); 6-((5-methyl-3-(6-methylpyridin-3-yl)isoxazol-4-yl) methoxy)-N-(tetrahydropyran-4-yl)pyridazine-3-carboxamide (IUPAC name); molecular weight, 409.44 gmol^−1^. **(B)** Concentration-response curves of alogabat in [^3^H]flumazenil competition-binding assays with membranes expressing different rat recombinant GABA_A_ receptor subtypes (*n* = 10–20/concentration). **(C)** Concentration-response curves of the effects of alogabat in HEK293 cells expressing different rat recombinant GABA_A_ receptor subtypes (*n* = 4–5/concentration). Data in the graphs are shown as the mean (symbols) ± SEM (error bars). Error bars smaller than the symbol size are not shown. **(D)** Isoguvacine concentration-response curves obtained in the presence and absence of alogabat in rat hippocampal slices. The points represent the mean values ± SEM of three slices. The sigmoidal curves were fit through these points. **(E)** The PPI of PS was measured in the absence (control) and presence of alogabat at 0.3 µM (*n* = 3 slices) and 1 µM (*n* = 6 slices). PS1 and PS2 are the responses induced by the first and second stimulus, respectively. The data are normalized to the amplitude of PS1 and presented as the mean ± SEM.

Alogabat [RO7017773; C_21_H_23_N_5_O_4_; 6-((5-methyl-3-(6-methylpyridin-3-yl)isoxazol-4-yl) methoxy)-N-(tetrahydropyran-4-yl)pyridazine-3-carboxamide (IUPAC name); molecular weight: 409.44 gmol^−1^] was synthesized at F.Hoffmann-La Roche AG, Switzerland [Figure 1A; see example 8 in Buettelmann et al., 2018]. The discovery of alogabat and the related chemical series will be disclosed in future publications. Diazepam, L-655,708, [^3^H]flumazenil, [^3^H]L-655,708, and [^3^H]RO0154513 were synthesized at F.Hoffmann-La Roche AG, Switzerland.

### 2.2 *In vitro* experiments

#### 2.2.1 GABA_A_ receptor radioligand binding assays

The affinity of alogabat for rat and human GABA_A_ receptors containing α5, α1, α2, and α3 subunits was measured by [^3^H]flumazenil competition-binding assays with membranes expressing different rat recombinant GABA_A_ receptor subtypes (*n* = 10–20/concentration) and for human α4 and α6 by [^3^H]RO0154513, as previously described (Ballard et al., 2009; Hipp et al., 2021).

#### 2.2.2 GABA_A_ receptor electrophysiological studies

Alogabat was tested for functional activity on four different cloned rat and human GABA_A_ receptor subtypes (Buettelmann et al., 2018), namely, α1β2γ2, α2β3γ2, α3β3γ2, and α5β3γ2. Ion currents were induced by GABA concentrations that evoked approximately 20% of the maximal response (*n* = 4–5/concentration). The following concentrations of GABA were used: 1 µM for α1β2γ2, 10 µM α2β3γ2, 10 µM for α3β3γ2, and 3 µM for α5β3γ2 receptor subtype.

Alogabat was also tested for functional activity on native GABA_A_-α5 receptors in rat hippocampal slices: modulation of isoguvacine-induced population spikes (Ballard et al., 2009) and paired-pulse inhibition (PPI) (Xie and Tietz, 1991). Population spikes (PSs) were recorded every 30 s by the stimulation of the Schaffer collaterals, and isoguvacine was applied consecutively and in increasing concentrations for at least 4 min to the slices (control). After the washout of isoguvacine, alogabat (300 nM) was added to the bath perfusion, and a new isoguvacine concentration-response curve was obtained (alogabat). The PS amplitudes were normalized to the control PS obtained before the first application of isoguvacine and were fitted individually for each slice (*n* = 3 slices).

The PPI of PS was measured in the absence (control) and presence of alogabat at 0.3 µM (*n* = 3 slices from one animal) and 1 µM (*n* = 6 slices from two animals). The paired-pulse protocol consisted of two stimuli timely spaced by 20 ms, which were applied at the Schaffer collaterals in rat hippocampal slices. We used a low number of animals based on our experience with the assay, which is highly robust and reproducible, and in alignment with our commitment to reducing the number of animals used.

#### 2.2.3 Selectivity screening

The selectivity of alogabat (10 µM) over more than 70 receptors was assessed by radioligand binding studies conducted using Eurofins Cerep SA (Celle Lévescault, France), following the methods described on www.eurofinsdiscoveryservices.com.

### 2.3 *In vivo* experiments

#### 2.3.1 Ethics approval

Experiments performed at F.Hoffmann-La Roche AG (Basel, Switzerland) complied with the Swiss federal and cantonal laws on animal research and AAALAC regulations and received prior approval from the Cantonal Veterinary Office. The EEG study was conducted at Brains Online (CA, United States) in accordance with the Guide for the Care and Use of Laboratory Animals (National Research Council 2011) after gaining approval from the Institutional Animal Care and Use Committee. The pentylenetetrazole (PTZ) and maximal electroshock (MES) tests were conducted at Vivocore Inc., Canada, which is a facility licensed by the Ontario Ministry of Agriculture, Food, and Rural Affairs and accredited by the Canadian Council on Animal Care. All *in vivo* studies are reported in compliance with the ARRIVE guidelines (Percie du Sert et al., 2020).

#### 2.3.2 Animals

All animals were group-housed in holding rooms at controlled temperature and humidity under a 12 h light/dark cycle and were allowed to acclimate for at least 7 days. Twenty-six male Wistar rats (Charles River, Germany; ∼160 g) and sixteen male Cntnap2^−/−^ mice (bred at Roche Basel; 18–22 g) were used in receptor occupancy studies. Thirty-two male Fischer rats (Charles River, Germany) were assessed in MRI experiments (∼250 g). Seventeen male Wistar rats (Envigo, United States) were assessed in EEG experiments. One hundred male BTBR mice (bred in Roche Basel) were assessed in grooming and open-field tests (n = 60, ∼30 g) and for digging behavior (n = 40, ∼30 g). Fifty-seven male Cntnap2^−/−^ mice (bred in Roche Basel; 5–6 weeks) and wildtype littermates (n = 15) were assessed in grooming and open-field tests. One hundred male Sprague–Dawley rats (Charles River, Canada) were used to evaluate antiepileptic effects in the PTZ (70–100 g, n = 40) and MES (<50 g n = 60) tests. Forty-eight male Sprague–Dawley rats (Charles River, France) were used in the rotarod test (∼170–200 g). One hundred and forty-one male Lister Hooded (LH) rats (Charles River, Germany) were used in the cognition tests: 5-choice serial reaction time (5-CSRT) task (n = 11, ∼400 g); continuous performance test (CPT; n = 11, ∼400 g); paired associates learning (PAL) task (n = 12, ∼400 g); context fear conditioning (n = 67, ∼250 g); and Morris water maze (n = 40, 350–400 g).

#### 2.3.3 Alogabat treatment

Alogabat was formulated in 2% hydroxypropyl cellulose in water (or 0.3% Tween 80 in saline for *in vivo* occupancy and grooming/digging experiments). The administration volume was 10 mL/kg (mice) and 5 mL/kg (rats). Each dose was expressed as the weight of the base. Alogabat was administered with a pre-treatment time of 30 min in mice and 60 min in rats (except for the EEG study), based on pharmacokinetic data indicating the time of maximal drug plasma concentration. Treatment groups were assigned randomly. Dose groups were provided as coded vials to allow blinded assessment during the conduct of each experiment. To minimize potential confounders regarding the time of day for behavioral testing, animals were split into separate cohorts (balanced for treatment groups) and tested during the same period.

#### 2.3.4 Receptor occupancy studies

##### 2.3.4.1 *In vivo* occupancy in rats

Male Wistar rats (n = 26, ∼160 g) were administered vehicle or alogabat (0.3, 1, 3, 10, 30, and 100 mg/kg, intraperitoneally [i.p.], n = 3/group) or the GABA_A_-α5 blocker L-655,708 at 10 mg/kg i.p. (n = 2). Fifteen minutes later, the GABA_A_-α5 radioligand [^3^H]RO0154513 was administered intravenously (i.v.) to assess RO (Ballard et al., 2009; Hipp et al., 2021). Non-specific binding was determined by pre-treatment with the established GABA_A_-α5 receptor blocker L-655,708 (10 mg/kg i.p.; n = 2).

##### 2.3.4.2 *Ex vivo* occupancy in Cntnap2^−/−^ mice

Cntnap2^−/−^ mice (Penagarikano et al., 2011) (n = 16, 18–22 g) were administered the vehicle or alogabat (30, 60, and 100 mg/kg) i.p. (n = 4/group), and RO was assessed in brain sections with the highly selective GABA_A_-α5 radioligand, [^3^H]L-655,708, 30 min post-administration.

#### 2.3.5 Pharmacological MRI in rats

Male Fischer rats (n = 32, ∼250 g) were administered vehicle or alogabat (3, 10, and 30 mg/kg) i.p. (n = 8/dose). phMRI was performed under medetomidine sedation in a small-animal scanner (4.7T/40 cm; Bruker BioSpin, Germany), and blood perfusion was analyzed according to previously published procedures (Bruns et al., 2009). Fifteen minutes after drug treatment, rats were initially anesthetized using isoflurane (4%) in the carrier gas composed of oxygen and air (1:5), supplied to the spontaneously breathing animals in an inhalation box. Upon the induction of anesthesia, a subcutaneous (s.c.) bolus injection of 0.2 mg/kg (1 mL/kg) of medetomidine prepared from 1:5 diluted Dormilan^®^ was administered, and the animal was transferred onto a rat cradle for inserting an s.c. catheter and starting continuous infusion of 1:10 diluted Dormilan^®^ (medetomidine) at a dose of 0.1 mg/kg/h (1 mL/kg/h). The head was immobilized in a stereotaxic frame. Respiratory rate, body temperature, and O_2_ and CO_2_ levels in inhaled and exhaled air were continuously monitored using a PowerLab Data Acquisition System (ADInstruments, Spechbach, Germany). Body temperature was maintained at 37°C with a feedback-regulated electric heating blanket. Immediately after the last imaging assessment, rats were euthanized by decapitation under anesthesia, and plasma samples were collected at approximately 40 min post-administration of alogabat. Images were processed and analyzed using in-house developed software written in IDL (RSI, Boulder, CO, United States) and MATLAB (The MathWorks Inc., Natick, MA, United States).

#### 2.3.6 EEG in rats

Male Wistar rats (n = 17) were surgically prepared with wireless implanted transmitters to enable simultaneous recording of EEG, hippocampal (CA3) local field potential (LFP), and EMG. Rats were anesthetized using isoflurane (2%, 800 mL/min O2). Bupivacaine/epinephrine was used for local analgesia, while Finadyne or carprofen was used for peri-/post-operative analgesia. Amoxicillin was used as an antibiotic. The animals were placed in a stereotaxic frame (Kopf instruments, United States). A subcutaneous pocket was created near the dorsal flank, into which a transmitter (F50-EEE, Data Sciences International, Physio Tel F50-EEE Small Animal CNS Telemetry; three bipolar channels, biopotential lead, outer diameter 0.3 mm) was inserted. The transmitter included three bipolar channels: channel 1 (EEG): two screws in the left hemisphere (frontal: 11 mm anterior and 2.5 mm lateral to bregma; central: 3 mm anterior and 3.5 mm lateral to lambda); channel 2 (LFP): a single wire electrode targeting the CA3 region of the right hippocampus; and channel 3 (EMG): electrodes placed in the neck muscle for recording EMG activity, aiding in sleep stage classification.

After a 2-week recovery, rats were administered the vehicle or alogabat (10 mg/kg) using a cross-over design (1-week interval). If the setup allowed, the positive control diazepam (2 mg/kg) was assessed. All drugs were administered i.v. via an implanted cannula so that brain exposure was expected within a few minutes of administration. EEG spectral power was analyzed quantitatively up to 1 h post-administration. Only animals with clean EEG recordings were included in the analysis. EEG data in the awake state were used for the analyses reported in this study. We first investigated the effects on power over predefined theta and beta power ranges, defined *a priori* as 6–10 Hz and 20–30 Hz, respectively, based on previous studies with GABAA drugs (Visser et al., 2003; van Lier et al., 2004). Then, we evaluated frequency-specific effects across the 2–128 Hz range.

#### 2.3.7 Grooming behavior and open-field test in BTBR and Cntnap2^−/−^ mice

Male BTBR mice (n = 60, ∼30 g) were pre-treated with the vehicle or alogabat at 30, 60, 90, and 120 mg/kg i.p. (n = 12/dose). Male Cntnap2^−/−^ mice (n = 57, 5–6 weeks) and wildtype littermates (n = 15) received sub-chronic treatment (8–10 days) with the vehicle or alogabat at 30, 60, and 100 mg/kg i.p. (n = 14–15/dose). Spontaneous locomotor activity was assessed in both groups of mice for 1 h in an open field (42 × 42 × 30.5 cm; 20 lux at the center of the arena) with a small amount of sawdust bedding. Movement of the animal was detected by interruptions in an array of photobeams from horizontally located infrared sources placed around the open field, measured using a VersaMax System (AccuScan Instruments Inc., Columbus, OH, United States). Activity was measured as the distance traveled (cm).

One week later, each mouse was placed into a type-II cage (21.5 × 15.5 × 13.0 cm; 40 lux) for 5 min habituation, and then the cumulative time (s) spent grooming during 10 min was recorded manually by a blinded observer. Distance traveled (cm) during the session was measured using a video tracking system (EthoVision, Noldus Information Technology, Netherlands). Plasma samples were collected from BTBR mice 1 week after testing and 30 min after treatment (*n* = 6/dose). Plasma samples were collected from Cntnap2^−/−^ mice 1 day later and 30 min after treatment (*n* = 4/dose).

#### 2.3.8 Digging behavior in BTBR mice

A separate group of male BTBR mice (n = 40, ∼30 g) were pre-treated with vehicle or alogabat at 30, 60, and 100 mg/kg i.p. (n = 10/dose) and were placed into a small circular dish (d = 10 cm) filled with fresh thick sawdust located in the middle of a clean type-II cage (21.5 × 15.5 × 13.0 cm; 40 lux). The cumulative time (s) spent digging over 5 min was recorded manually by a blinded observer. The distance traveled (cm) and speed were also measured in the arena, outside the dish (EthoVision, Noldus Information Technology, Netherlands). Plasma samples were collected immediately after the digging test, i.e., 35–40 min post-administration (*n* = 3/dose).

#### 2.3.9 Evaluation of antiepileptic effects in rats

Vehicle or alogabat (5, 15, or 30 mg/kg) was administered p.o. (n = 8/dose) and assessed for seizures induced by (1) subcutaneous (s.c.) PTZ and (2) the maximum electrical stimulus (MES) model in male Sprague–Dawley rats (PTZ: 70–100 g, n = 40; MES: <50 g n = 60, Charles River, Canada). Diazepam (synthesized at F.Hoffmann-La Roche AG) was included in each experiment as a positive control and administered at 3 mg/kg i.p., 30 min prior to testing. Immediately following testing, a terminal blood sample was collected under anesthesia.

##### 2.3.9.1 Pentylenetetrazole test

Following the designated pre-treatment time, PTZ was administered at 75 mg/kg s.c. Animals were transferred to a single cage and monitored closely for the onset of clonic seizure, and the percentage of each group showing seizures was recorded. Protection was defined as the complete absence of clonic seizure, including forelimb clonus, over the 30 min observation period.

##### 2.3.9.2 Maximal electroshock test

Following the designated pre-treatment time, rats received maximal electroshock (MES: 150 mA, 0.2 s duration, 60 Hz) via corneal electrodes moistened with saline (shock simulator type 221; Harvard Apparatus). Due to inconsistency in responses by rats to this type of stimuli, all test animals (n = 60) were subjected to two screening sessions on two separate days, and 40 rats were selected based on their reliability to have tonic seizures. Drug testing was conducted on the third day, during which the rats received the standard maximal electroshock, and the presence or absence of a tonic seizure was recorded and expressed as the percentage of animals in each group that showed seizures. Protection was defined as the absence of a full tonic seizure within 15 s of stimulus delivery.

#### 2.3.10 Combination with diazepam on rotarod performance in rats

Forty-eight male Sprague–Dawley rats (∼170–200 g) were pre-trained to the rotarod (47750 Rota-Rod NG, Ugo Basile) at 8 rpm for 120 s. One day later, rats were administered alogabat (or vehicle) p.o., 30 min prior to diazepam or vehicle i.p., and they were assessed on the rotarod 30 min later. Each rat was given three attempts to remain on the rotarod, with a maximum duration of 120 s per attempt. Treatment groups (n = 8/group) consisted of alogabat alone (30 mg/kg), diazepam alone (5 mg/kg), or alogabat at 3, 10, and 30 mg/kg in combination with diazepam (5 mg/kg). The maximum time (s) spent on the rotarod was recorded. After the completion of rotarod testing, animals were euthanized under isoflurane anesthesia for plasma sample collection at approximately 70 min post-administration of alogabat.

#### 2.3.11 Cognition testing in rats

##### 2.3.11.1 Operant and touchscreen cognition tests

Separate groups of male Lister Hooded (LH) rats (∼400 g) were pre-trained in the operant and touch screen tests, as described in Supplementary Material. The 5-CSRT task (n = 11) is a test of attention. The primary parameter is the total percentage of correct responses. Continuous performance test (CPT; n = 11) assesses sustained attention. The primary parameter is the discrimination sensitivity index (D prime), which is a ratio based on the rate at which rats make correct and incorrect responses. The paired associates learning (PAL) task (n = 12) assesses visual–spatial memory. The primary parameter is the total percentage of correct responses. A Latin-squares design was used to assess the vehicle and doses of alogabat (3, 10, and 30 mg/kg p.o.). Rats were tested twice weekly with at least a 2-day interval between test sessions. Rats were trained between test days to maintain baseline performance.

##### 2.3.11.2 Context fear conditioning

Two cohorts of male LH rats (∼250 g; n = 67) were assessed for context fear conditioning (CFC) in two experiments: (1) vehicle or alogabat at 10 and 30 mg/kg (n = 9/dose); (2) vehicle or alogabat at 1, 3, and 10 mg/kg (n = 10/dose) administered p.o., 60 min prior to the acquisition session. Rats were placed into a novel test chamber (45 cm × 45 cm × 46.5 cm) with a grid floor and clear plastic walls (TSE-systems GmbH, Germany). During the acquisition session, a 2-min habituation phase was followed by the delivery of a mild foot shock (0.8 mA for 1 s) applied through the grid floor. Following a 1 min interval, a second foot shock was delivered. The animals remained in the chambers for a further 30 s. One day later, the animals were returned to the same test chamber for a 5-min period without any foot shock. Rodents respond to danger in a species-specific manner by freezing, i.e., the animals will withhold all movement, except for respiration, to avoid detection. The amount of freezing behavior was measured by the computer as the time spent immobile (minimum threshold of 1 s) and was expressed as the percentage of time spent freezing during the 5-min session. Following CFC, plasma samples were collected 1–2 days later from rats pre-treated with the same dose at 60 min post-administration (*n* = 4/dose).

##### 2.3.11.3 Morris water maze

Male LH rats (350–400 g) were pre-treated with vehicle or 10 or 30 mg/kg alogabat p.o. (n = 10/dose) once daily during cued acquisition (visible platform; three trials per day for 2 days), followed by a 2-day interval and then spatial acquisition (hidden platform; three trials per day for 4 days) in the Morris water maze (MWM; computer tracking system HVS Image Ltd., United Kingdom) (Ballard et al., 2005). Twenty-four hours later, the platform was removed, and the rats were pre-treated with vehicle or alogabat and allowed to swim in the maze for 60 s (probe trial) to assess the retention/spatial memory of the platform position, expressed as the percentage of distance traveled in each quadrant. For the MWM, plasma samples were collected immediately after the last trial on the 12th day of treatment at approximately 70 min post-administration (*n* = 4–5/dose).

#### 2.3.12 Plasma sampling and analysis

Rodents were deeply anesthetized with 5% isoflurane, and blood was collected following decapitation with a guillotine or via cardiac puncture (PTZ and MES). During EEG experiments, blood was collected from a cannula in the right femoral artery. Blood samples were centrifuged in EDTA-coated Eppendorf tubes (4,000 rpm, 4°C, 5 min), and plasma was collected, immediately frozen, and stored at −80°C. Samples were analyzed for the concentration of alogabat using qualified liquid chromatography coupled to tandem mass spectrometry (LC-MS/MS) assays.

#### 2.3.13 Statistical analysis

Sample sizes were determined based on historical data for each experiment within each laboratory.

##### 2.3.13.1 EEG

Linear mixed-effects models (LMMs) were used since these can account for incomplete datasets, which was the case in this study. Specific contrasts were derived and tested using t-tests within the model using Satterthwaite approximation for degrees of freedom. Models were derived for each compound individually: Y ∼ COMPOUND + (1|ANIMAL). The significance of differences in the peak frequency was assessed using random permutation tests (10,000).

##### 2.3.13.2 phMRI

Analysis was performed with JMP (SAS Institute Inc., Cary, United States) and MATLAB (The MathWorks Inc., Natick, MA, United States). Global (whole-brain) absolute-perfusion values from the vehicle and the three dose groups were tested for a linear trend across the four doses using a one-way ANOVA framework. The same analysis was applied ROI-wise to the normalized-perfusion values. Multiple testing across the single ROIs was accounted for by controlling the false discovery rate (FDR) at 10% using the Benjamini–Hochberg approach. To also obtain an overall activation–strength metric, normalized-perfusion values of each dose group were compared ROI-wise to those of the vehicle group using Welch’s t-test without multiple-testing correction. The number of significantly modulated ROIs at each dose was then taken as a measure of “pattern strength.” The mean and the upper 95% confidence limit of the corresponding chance levels were estimated via random group-label permutations (100,000 runs per group). Significance levels of the actual pattern strengths were determined based on the distributions obtained through the permutation procedure.

##### 2.3.13.3 Behavior

All statistical analyses were performed using Prism (GraphPad software). One-factor ANOVA was used for between-group comparison (i.e., dose), with repeated measures for within-subject comparison (i.e., time bins and platform quadrants). Dunnett’s test was used for *post hoc* comparisons. Student’s unpaired two-tailed t-test was used when two groups were compared. The chi-square test was used to compare the percentage of groups. Rotarod data were analyzed using a non-parametric Kruskal–Wallis test, followed by Dunn’s multiple comparison test. Statistical significance was predetermined as *p* < 0.05.

## 3 Results

### 3.1 Binding affinity for GABA_A_ receptors containing α5 and other α subunits

The aim of these radioligand binding studies was to determine the binding selectivity of alogabat at the rat and human GABA_A_ receptors containing α5 compared to α1, α2, and α3 subunits. Alogabat had a high affinity for GABA_A_-α5β3γ2 receptors in rats (Ki 7.9 nM), with approximately 37-, 26-, and 18-fold selectivity compared to GABA_A_ α1β3γ2, α2β3γ2, and α3β3γ2, respectively (Figure 1B; see Supplementary Table S1). Alogabat had a similar pharmacological profile in human recombinant GABA_A_ receptor subtypes (see Supplementary Table S1).

### 3.2 Functional activity at recombinant and native GABA_A_ receptors

We undertook electrophysiological studies in HEK293 cells and oocytes to assess the functional activity of alogabat on cloned rat and human GABA_A_ receptor subtypes containing α5, α1, α2, and α3 subunits. Alogabat enhanced GABA-evoked responses of rat and human α5-containing receptors by 167% (Figure 1C) and 72% (see Supplementary Table S2), respectively. The maximum amount of potentiation was slightly lower at human receptors. This may be due to the different expression systems used, i.e., HEK293 cells for rat receptors and *Xenopus* oocytes for human receptors. At the highest concentrations tested, alogabat potentiated other receptor subtypes to a much lesser extent than α5-containing receptors. The potency of alogabat was comparable between GABA_A_-α5 receptors containing either γ2 or γ3 subunits, i.e., GABA_A_-α5β3γ2 and GABA_A_-α5β3γ3 receptors (see Supplementary Figure S1).

At native GABA_A_ receptors in rat hippocampal slices, alogabat positively modulated GABAergic inhibition of CA1 pyramidal cells, as shown by a leftward shift of the concentration-dependent inhibition of the population spike by isoguvacine (Figure 1D) and enhanced paired-pulse inhibition of the population spike during field potential recordings (Figure 1E). Together, these results confirm the binding selectivity of alogabat and demonstrate that alogabat is a functionally selective PAM of GABA-evoked responses.

### 3.3 Selectivity data

We assessed alogabat in radioligand binding studies with more than 70 receptors and showed that alogabat at 10 µM had over 917-fold binding selectivity for the GABA_A_-α5β3γ2 receptor subtype against all other targets tested (see Supplementary Table S3).

### 3.4 Receptor occupancy in Wistar rats and Cntnap2^−/−^ mice

**FIGURE 2:**
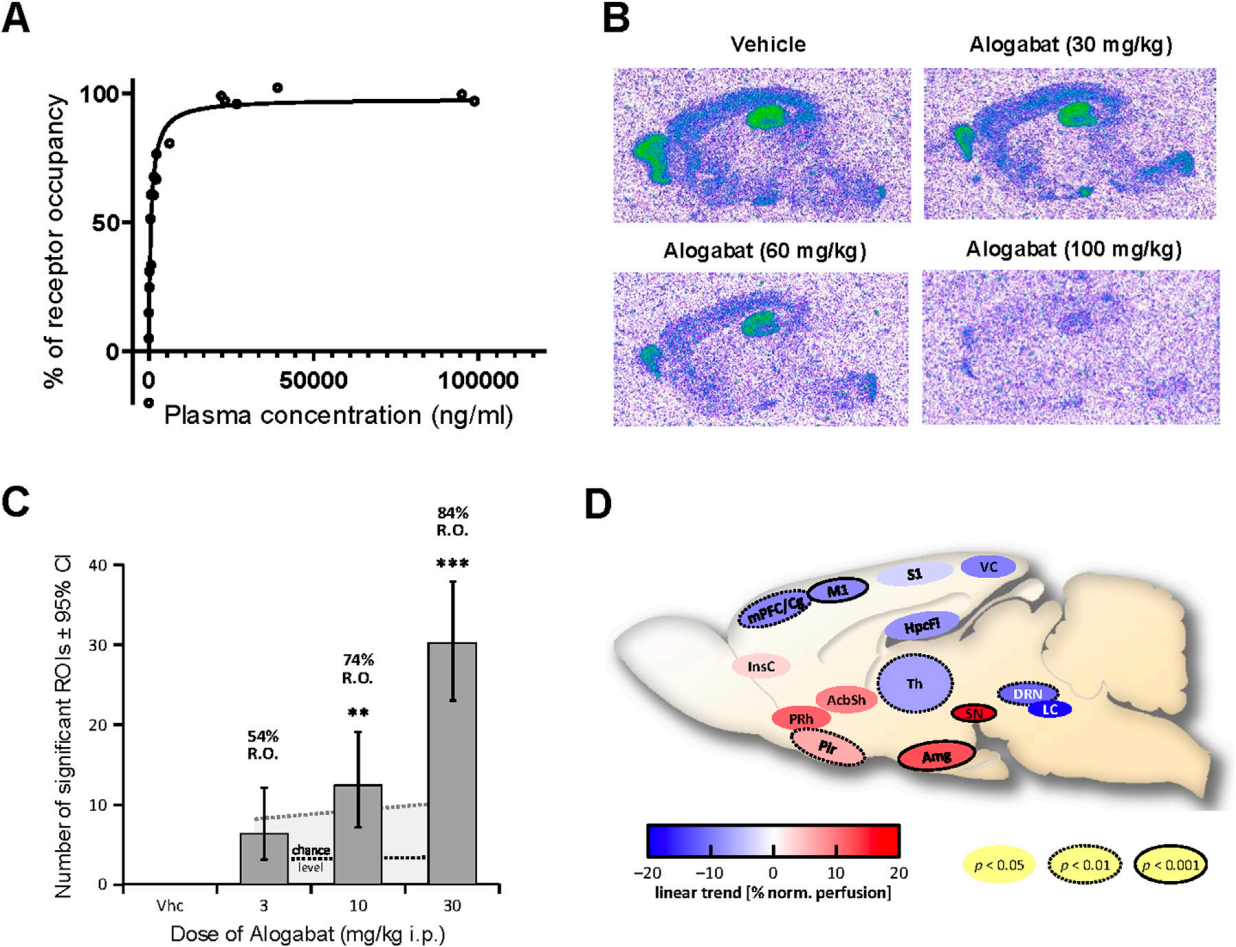
Receptor occupancy and phMRI signature of alogabat following acute administration. **(A)** Concentration-dependent blockade of [^3^H]RO0154513 binding by alogabat in Wistar rats: with increasing concentrations from the different doses (0.3–100 mg/kg i.p., n = 3/dose) of alogabat (vehicle = without pre-treatment, n = 3). **(B)** Representative autoradiograms of alogabat *ex vivo* occupancy in Cntnap2^−/−^ mice via *in vitro* incubation of the highly selective GABA_A_-α5 receptor radioligand [^3^H]L-655,708 to sagittal brain sections. The Cntnap2−/− mice were treated with vehicle and 30, 60, or 100 mg/kg i.p. alogabat (n = 4/dose). **(C)** Trend of normalized-perfusion response pattern strength increasing with dose, given as numbers (± 95% confidence intervals) of significantly (de-)activated regions of interest (ROIs) following the administration of alogabat (n = 8/dose) to Fischer (F344) rats under medetomidine sedation. “Chance level” = mean (black dotted line) and 95th percentile (gray dotted line) derived from the permutation test. Significant deviations in the number of modulated ROIs from the chance level are denoted by *p < 0.05, **p < 0.01, and ***p < 0.001 (uncorrected). RO is the calculated receptor occupancy for GABA_A_-α5 from measured plasma concentrations at each dose. **(D)** Sagittal schematic of rat brain showing ROIs with a significant dose-dependent modulation (linear trend of normalized perfusion). ROIs passing false discovery rate control at 10% are shown, with ROI outlines indicating uncorrected significance level (no outline, *p* < 0.05; dotted outline, *p* < 0.01; and solid outline, *p* < 0.001). ROI abbreviations: AcbSh, nucleus accumbens shell; Amg, amygdala; DRN, dorsal raphe nucleus; HpcFi, fimbria hippocampi; InsC, insular cortex; LC, locus coeruleus; M1, primary motor cortex; mPFC/Cg, medial prefrontal, cingulate cortex; Pir, piriform (entorhinal) cortex; PRh, perirhinal cortex; S1, primary somatosensory cortex; SN, substantia nigra; Th, thalamus; VC, visual cortex.

The main objective of these studies was to visualize and quantify GABA_A_-α5 RO by alogabat. Alogabat decreased the specific binding of the GABA_A_-α5 receptor-specific radioligand [^3^H]RO0154513 in the hippocampus of rats in a dose-dependent manner. The highest dose of alogabat (100 mg/kg i.p.) reduced specific binding by 100%, i.e., to the same binding level as that of 10 mg/kg L-655,708 (Figure 2A). A total plasma concentration of 669 ng/mL and a total brain concentration of 208 ng/g were required to produce half-maximal GABA_A_-α5 RO (EC_50_) in the hippocampus in rats. In Cntnap2^−/−^ mice, estimated GABA_A_-α5 RO based on the *ex vivo* study with [^3^H]L-655,708 was 43%, 46%, and 71% at doses of 30, 60, and 100 mg/kg i.p., respectively (Figure 2B). In addition, the rat occupancy curve was used to calculate RO related to plasma exposure for all *in vivo* experiments described in this manuscript below (also refer to Supplementary Tables S4–S5).

### 3.5 Pharmacological MRI signature in rats

The purpose of this study was to evaluate the effects of alogabat on regional neural activity. Blood perfusion, measured quantitatively by phMRI, was taken as a proxy for neural activity. The administration of a single dose of alogabat at 3–30 mg/kg i.p. in rats resulted in moderate but consistent and dose-dependent changes in neural activity patterns compared to treatment with the vehicle (Figure 2C). Significant dose-dependent modulation of normalized perfusion by alogabat was observed in limbic brain regions, i.e., the nucleus accumbens (shell), amygdala, and hippocampus (fimbria); in several cortical brain regions, the thalamus and brainstem nuclei; and in the substantia nigra (Figure 2D). A linear dose effect was also observed on whole-brain absolute perfusion. Alogabat target engagement in brain tissue ranged from 54% to 84% RO.

### 3.6 EEG signature in rats

**FIGURE 3:**
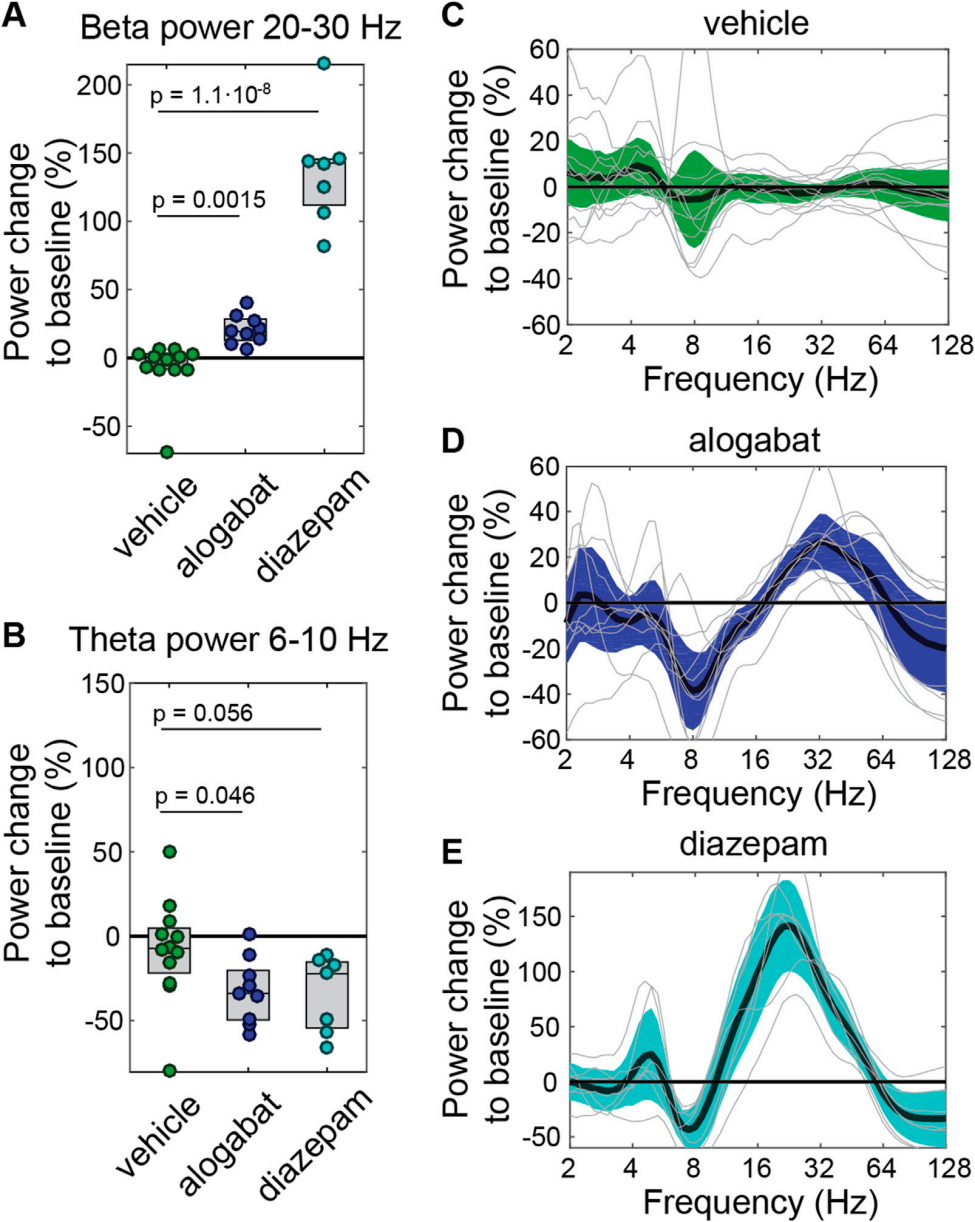
Pharmacodynamic effect of alogabat on EEG spectral power. **(A,B)** Modulations of vehicle (*n* = 12), alogabat 10 mg/kg (*n* = 9), and diazepam 2 mg/kg (*n* = 7, positive control) in the beta (20–30 Hz) and theta (6–10 Hz) power. **(C–E)** Effects of individual compounds and vehicle on the EEG power spectrum. Each individual recording is displayed as a light gray line, and the group average and its 95% confidence intervals are shown as colored bands. For all panels, power averaged over 0.2–1 h post dosing is shown relative to pre-dosing baseline average over −1 to −0.2 h before dosing. Statistics: *p < 0.05 versus vehicle (linear mixed-effects models followed by t-tests).

The aim of this study was to determine whether alogabat induces a characteristic EEG signature that could be used as a translational pharmacodynamic marker. As a first step and based on previous findings on the EEG effects of GABA_A_ drugs (van Lier et al., 2004; Visser et al., 2003; Friedman et al., 1992), we investigated EEG spectral power change in the theta (6–10 Hz) and beta (20–30 Hz) frequency bands. Alogabat, administered as 10 mg/kg i.v. (5 min infusion), significantly increased EEG beta-band activity by 21% ± 11% relative to the baseline (mean ± SD) and decreased theta-band activity by 32% ± 19% (Figures 3A,B). Frequency-resolved changes confirmed a decrease in theta power (approximately 6–10 Hz) and revealed broader beta and gamma modulation (approximately 20–50 Hz) (Figures 3C–E). The calculated RO after the application of alogabat was 77%–85% for GABA_A_-α5 (see Supplementary Table S6). The overall spectral pattern was qualitatively similar to that of diazepam; however, the peak frequency in the beta frequency range was higher for alogabat (32 Hz for alogabat vs. 22.6 Hz for diazepam; *p* = 0.006, random permutation test), and its amplitude was greater for diazepam (142% ± 44% increase for diazepam vs. 27% ± 16% increase for alogabat; *p* < 0.001). This is likely related to the high binding and functional selectivity of alogabat for α5-containing GABA_A_ receptors compared to the non-selective profile of diazepam.

### 3.7 Repetitive behaviors in mouse models relevant to ASD

The inbred mouse strain BTBR and the Cntnap2^−/−^ mouse show abnormalities in core ASD behavioral domains, such as repetitive behaviors. Self-grooming and digging are more elevated in the BTBR mouse than in other strains, i.e., C57BL/6J (McFarlane et al., 2008), and Cntnap2^−/−^ mice show more elevated grooming than their wildtype littermates (Penagarikano et al., 2011). The aim of the following experiments was to determine whether alogabat has a specific effect on repetitive behaviors independent of potential effects on overall locomotor activity. We assessed the effects of subchronic treatment on Cntnap2^−/−^ mice to exclude potential tolerance to the effects of alogabat.

#### 3.7.1 Open field in BTBR and Cntnap2^−/−^ mice

**FIGURE 4:**
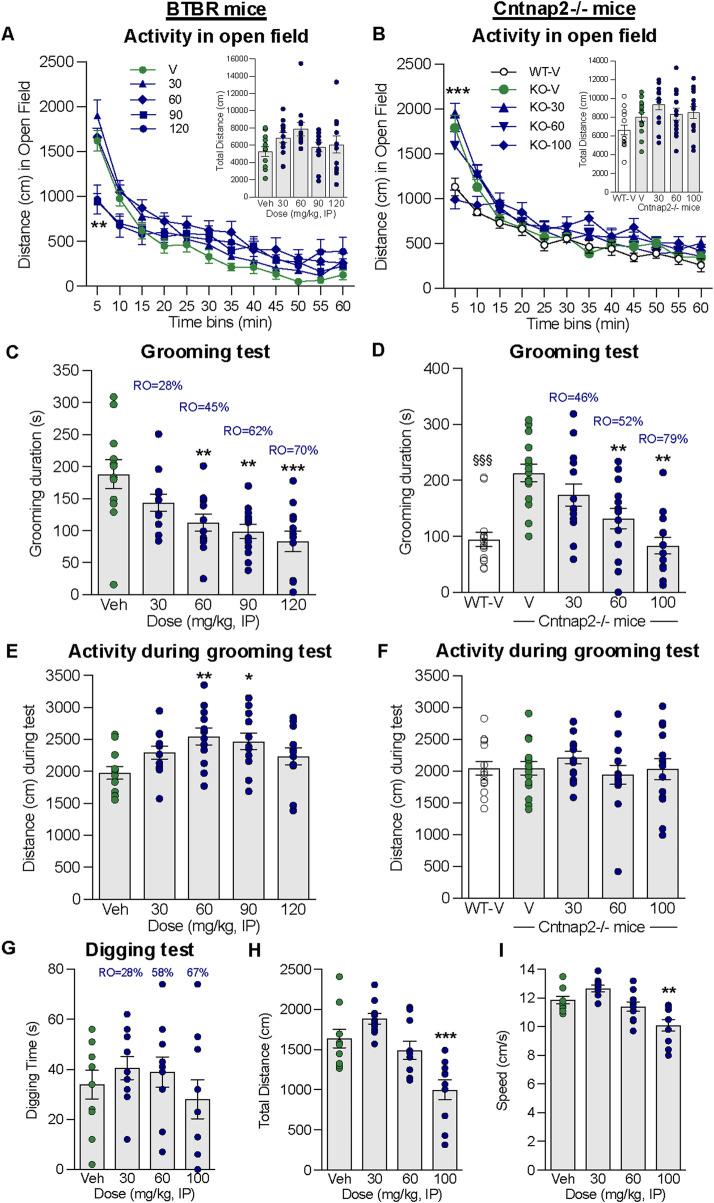
Effect of alogabat on repetitive behaviors in BTBR and Cntnap2^−/−^ mice. **(A)** Alogabat was administered i.p. 30 min prior to the assessment of locomotor activity in an open-field test in BTBR mice: distance traveled (cm) per 5 min time bin (*line graph*) and in 1 h total (*bar chart inset*). **(B)** Alogabat was administered i.p. once daily for 2–3 days, and on the test day, it was administered 30 min prior to testing Cntnap2^−/−^ mice in an open field: distance traveled (cm) per 5 min time bin (*line graph*) and in 1 h total (*bar chart insert*). In BTBR mice, on a separate test day, alogabat was administered i.p. 30 min prior to the grooming test: **(C)** duration (s) of grooming behavior and **(E)** distance (cm) traveled during the 10 min period in the test chamber. In Cntnap2^−/−^ mice, alogabat was administered i.p. once daily for 8–10 days, and on the test day, it was administered 30 min prior to the grooming test: **(D)** duration (s) of grooming behavior and **(F)** distance traveled during the 10 min period in the test chamber. Data are presented as individual points (circles) and mean ± SEM (BTBR: *n* = 12/group; Cntnap2^−/−^: *n* = 14–15/group). Statistics: *p < 0.05, **p < 0.01, and ***p < 0.001 vs. vehicle (Veh)-treated BTBR or Cntnap2^−/−^ mice. §§§*p* < 0.001 vehicle-treated wildtype (WT-V) mice vs. vehicle (V)-treated Cntnap2^−/−^ mice. Alogabat was administered i.p. 30 min prior to the assessment of digging behavior in BTBR mice: **(G)** duration (s) of digging behavior, **(H)** distance traveled (cm), and **(I)** speed (cm/s) during the 5 min period in the test chamber. Data are presented as individual points (circles) and mean ± SEM (*n* = 12/group). Statistics: **p < 0.01 and ***p < 0.001 vs. vehicle-treated BTBR mice. RO is the calculated receptor occupancy for GABA_A_-α5 from the measured plasma concentrations at each dose.

In BTBR mice, acute administration of alogabat (30–120 mg/kg i.p.) had no effect on the total activity over 1 h measured in an open field [F(4,55) = 2.04], although during the first 10 min, alogabat at 90 and 120 mg/kg significantly reduced the distance traveled [F(4,55) = 9.623, *p* < 0.001, Figure 4A]. There was also no effect of sub-chronic alogabat (30–100 mg/kg i.p.) on the total activity over 1 h in Cntnap2^−/−^ mice [F(3,53) = 0.98]. During the first 5 min, 100 mg/kg reduced the distance traveled in Cntnap2^−/−^ mice to the same level as vehicle-treated wildtype mice (Figure 4B). Alogabat did not induce a major impairment of locomotor activity in the open field.

#### 3.7.2 Grooming behavior in BTBR and Cntnap2^−/−^ mice

Acute administration of alogabat in BTBR mice dose-dependently attenuated grooming behavior at 60, 90, and 120 mg/kg i.p. [F(4,55) = 6.946, *p* = 0.0001, Figure 4C] without reducing locomotor activity in the same test arena (Figure 4E). In contrast, doses of 60 and 90 mg/kg increased the distance traveled [F(4,55) = 3.454, *p* < 0.05]. Alogabat had a specific effect on grooming behavior at plasma concentrations corresponding to calculated GABA_A_-α5 RO levels of 45%–70% (see Supplementary Table S4).

Sub-chronic administration of alogabat in Cntnap2^−/−^ mice attenuated grooming behavior at 60 and 100 mg/kg i.p. [F(3,53) = 10.57, *p* < 0.0001, Figure 4D], with no effect on locomotor activity measured in the same test arena [F(3,53) = 0.74, Figure 4F]. Alogabat had a specific effect on repetitive grooming behavior at plasma concentrations that correspond with calculated GABA_A_-α5 RO of 52%–79% (see Supplementary Table S4).

#### 3.7.3 Digging behavior in BTBR mice

There was no effect of acute alogabat administration on digging behavior at doses up to 100 mg/kg i.p. [F(3,36) = 0.8, Figure 4G], corresponding to a calculated GABA_A_-α5 RO level of 67% (see Supplementary Table S4). Alogabat at 100 mg/kg reduced the distance traveled [F(3,36) = 12.1, *p* < 0.001, Figure 4H] and speed [F(3,36) = 11.5, *p* < 0.001, Figure 4I] in the same arena.

### 3.8 Anticonvulsant activity in rats

**FIGURE 5:**
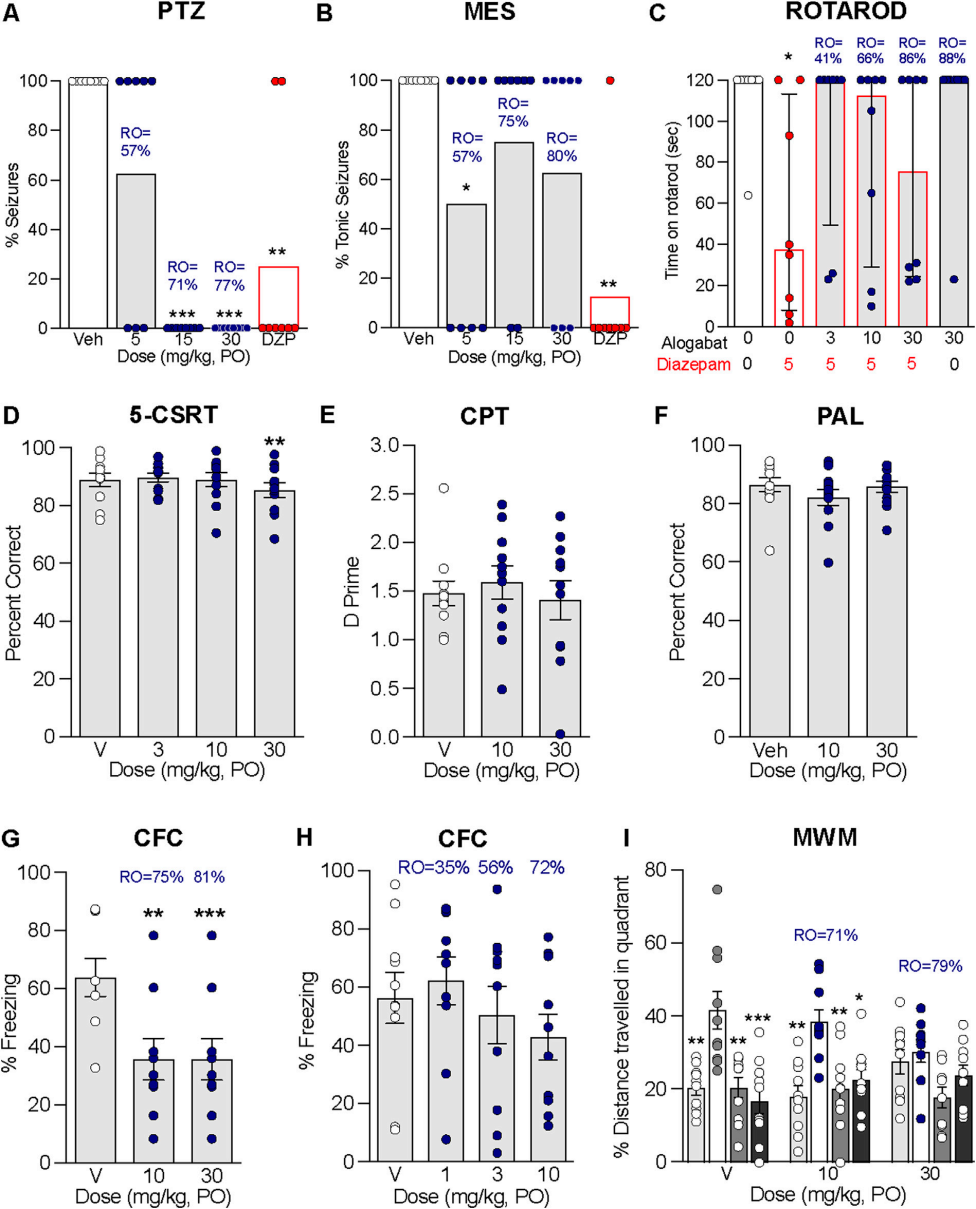
Evaluation of alogabat for effects on anticonvulsant, motor, and cognitive tests. Alogabat was administered p.o. 60 min prior to the following behavior tests. Assessment of potential anticonvulsant activity in **(A)** PTZ and **(B)** MES tests in rats. Diazepam (DZP) at 3 mg/kg i.p. was included as a positive control. Data are presented as individual points (circles) and the percentage of rats per group showing seizures (*n* = 8/group). **(C)** Time (s) spent on the rotarod at 16 rpm in rats administered alogabat alone (30 mg/kg, gray bar with black outline) or alogabat at 3, 10, and 30 mg/kg co-administered with diazepam at 5 mg/kg i.p. (gray bars with red outline) compared with vehicle (open bar) or diazepam (open bar with red outline). Data are presented as individual points (circles) and median ± interquartile range (n = 8/group). Assessment of cognition in rats: **(D)** attention in the 5-choice serial reaction time (5-CSRT) task; **(E)** sustained attention in the touchscreen continuous performance test (CPT); **(F)** visual–spatial memory in the touchscreen PAL task. CFC at **(G)** 10 and 30 mg/kg; **(H)** 1, 3, and 10 mg/kg. Spatial learning and memory in the MWM: alogabat was administered once daily for 11 days prior to the probe trial. **(I)** Percentage of distance traveled in all four quadrants: vehicle; 10 mg/kg; 30 mg/kg. Data are presented as individual points (circles) and mean ± SEM for PAL (*n* = 12/dose; within-subjects), 5-CSRT and CPT (*n* = 11/dose, within-subjects), CFC (*n* = 9–10/group), and MWM (*n* = 10/group). Statistics: *p < 0.05, **p < 0.01, and ***p < 0.001 vs. vehicle (Veh or 0)-treated rats; MWM statistics: *p < 0.05, **p < 0.01, and ***p < 0.001 vs. percentage of distance in the platform quadrant. RO is the calculated receptor occupancy of alogabat for GABA_A_-α5 from measured plasma concentrations at each dose.

The purpose of these experiments was to evaluate the antiepileptic properties of alogabat against seizures induced by PTZ and the MES model. Alogabat administered in combination with a convulsant dose of PTZ completely blocked seizures at 15 and 30 mg/kg (Figure 5A), which corresponds to calculated GABA_A_-α5 RO levels of 71% and 77% (see Supplementary Table S5). Meanwhile, in the MES test, alogabat at 5 mg/kg (57% RO, see Supplementary Table S5) showed a significant reduction in the percent of rats that had tonic seizures (Figure 5B); there was only a tendency for reduction at 15 mg/kg (*p* = 0.1) and 30 mg/kg (*p* = 0.06). The positive control, diazepam, significantly reduced the percentage of rats that had seizures in both the PTZ and MES tests.

### 3.9 Combination with diazepam on rotarod performance in rats

Classical benzodiazepines, such as diazepam, are known to induce sedation and ataxia, which can be measured by the rotarod task in rodents. The aim of this experiment was to determine whether alogabat affects rotarod performance when administered alone and, second, whether alogabat modulates the sedative/motor effect of diazepam on rotarod performance when given in combination. Diazepam at 5 mg/kg i.p. significantly impaired rotarod performance. Alogabat (3–30 mg/kg p.o.) co-administered with diazepam did not worsen the effects of diazepam on rotarod performance (Figure 5C). Alogabat administered alone at 30 mg/kg p.o. did not differ from vehicle-treated rats, corresponding to a calculated GABA_A_-α5 RO level of 88% (see Supplementary Table S5).

### 3.10 Evaluation of cognition in rats

Since non-selective GABA_A_ PAMs, such as diazepam, have been shown to impair cognition (Ballard et al., 2005), the aim of the following experiments was to study the potential effects of acute administration of alogabat on cognition in rats. This included attention in the 5-CSRT task and associative (fear) memory in CFC. We also assessed visual–spatial memory (PAL task) and attention (CPT) in a touchscreen system, which provides a translational approach for assessing cognition (Hvoslef-Eide et al., 2016). Chronic treatment with alogabat was also assessed on spatial learning and memory in the MWM.

In the 5-CSRT task, alogabat at 30 mg/kg induced a small but statistically significant reduction in percent correct (3%; within-subjects comparison; F(3,30) = 5.6, *p* < 0.01; Figure 5D). However, alogabat did not impair attention in CPT (F(2,20) = 0.3; Figure 5E) or visual–spatial memory in the PAL task (F(2,22) = 2.6; Figure 5F).

In the CFC test, alogabat was administered before the acquisition session to determine whether there was an effect on learning the association between foot shock and the surrounding context. In the first experiment, alogabat impaired CFC at 10 and 30 mg/kg, corresponding to calculated GABA_A_-α5 RO levels of 75% and 81% (F(2,24) = 10.99, *p* < 0.001; Figure 5G). A second experiment was undertaken to explore lower doses, and it showed that alogabat did not significantly impair CFC at 1, 3, and 10 mg/kg (F(3,36) = 0.9; Figure 5H), corresponding to the calculated GABA_A_-α5 RO levels of 35%, 56%, and 72%. This suggests that the dose of 10 mg/kg is approximately the threshold level for the impairment of CFC and is likely dependent on the level of RO achieved per subject.

In the MWM test, chronic administration of alogabat at 10 and 30 mg/kg did not significantly impair the acquisition of the hidden platform position (see Supplementary Figure S2). Twenty-four hours later, the platform was removed, and the rats were allowed to swim in the maze for 60 s (probe trial) to assess memory-retention of the platform position. The groups receiving vehicle [F(3,27) = 8, *p* < 0.001] and 10 mg/kg [F(3,27) = 7, *p* < 0.01] had learned the spatial position of the platform, as shown by their preference for the platform quadrant (Figure 5I). However, rats that received 30 mg/kg alogabat showed impaired spatial memory of the platform position during the probe trial [F(3,27) = 3, p = 0.08; Figure 5I], and GABA_A_-α5 RO was estimated to be 79%. In summary, alogabat impaired context and spatial memory at GABA_A_-α5 RO above 70%.

## 4 Discussion

### 4.1 *In vitro* and *in vivo* characterization of alogabat

Alogabat was shown to be a potent and functionally selective GABA_A_-α5 receptor PAM *in vitro*. The functional activity of alogabat was confirmed at native GABA_A_ receptors in rat hippocampal slices. *In vivo* binding experiments demonstrated dose-dependent target engagement of alogabat in rats and Cntnap2^−/−^ mice. The highest specific binding was observed in the hippocampus, which is consistent with the known distribution of the GABA_A_-α5 receptor subtype in rodents (Pirker et al., 2000). phMRI studies in rats demonstrated dose-dependent perfusion changes, indicating circuit modulation by alogabat. There were consistent changes in the neural activity pattern across dose groups, with modulation of neural activity in, among others, the medial prefrontal cortex, cingulate cortex, motor cortex, perirhinal cortex, nucleus accumbens shell, amygdala, hippocampus fimbria, and substantia nigra.

### 4.2 EEG signature

Acute administration of alogabat in rats induced an EEG signature characterized by a power decrease in theta and an increase in the beta frequency range, which was qualitatively similar to the EEG signature of non-selective GABA_A_ PAMs such as diazepam (van Lier et al., 2004; Visser et al., 2003; Friedman et al., 1992). The lower magnitude of induced EEG beta-band power by alogabat compared to diazepam is likely attributed to its α5-selectivity, resulting in an overall lower magnitude of GABA_A_ receptor modulation. This EEG signature of alogabat is qualitatively a mirror image of the EEG signature of basmisanil, a highly selective GABA_A_-α5 negative allosteric modulator (NAM), which we recently found to induce an increase in the theta power and a decrease in the beta-band EEG power in healthy volunteers (Hipp et al., 2021). Furthermore, the EEG signature of alogabat is in agreement with the link between beta-band activity and single-nucleotide polymorphisms (SNPs) in GABA_A_ receptor genes (Porjesz et al., 2002; Smit et al., 2018) and NDDs (Dup15q syndrome, AS) involving gene copy number variations of *GABRB3, GABRA5*, and *GABRG3* (Frohlich et al., 2019a; Frohlich et al., 2019b). GABA_A_ receptor-related changes in beta-band activity are believed to reflect the modulation of recurrent excitatory–inhibitory feedback loops in the cortical tissue (Whittington et al., 2000; Traub and Jefferys, 1999). For the administered doses, the calculated receptor occupancies were in the range of 77%–84% for α5-containing receptors and 22%–34% for α1-containing receptors (with different PAM effects (E_max_) at α5 vs. α1). Thus, at the studied doses, alogabat has some effect on α1-containing receptors, and further work is needed to systematically study lower doses of alogabat, including those <70% RO without the impairment of cognition in WT rats, to confirm whether the modulation of α5-containing receptors alone is sufficient to induce the described EEG effect. Nonetheless, EEG theta- and beta-band power are promising candidates for translational mechanistic biomarkers of GABA_A_-α5 receptor function and could be used in the further development of alogabat.

### 4.3 Behavioral effects of alogabat in animal models relevant to ASD

We assessed the effects of alogabat in two different mouse models relevant to ASD, one phenotypic and one genetic. Acute administration of alogabat significantly and dose-dependently attenuated excessive grooming, but not digging, behavior in BTBR mice. Sub-chronic administration of alogabat significantly attenuated elevated grooming behavior in Cntnap2^−/−^ mice. Efficacy in these different models was linked to GABA_A_-α5 RO above 50%. Despite initial findings of a transiently decreased activity in an open-field test, alogabat increased the activity of BTBR mice and had no effect on the activity of Cntnap2^−/−^ mice during the grooming test, supporting the specific effect of alogabat on repetitive grooming. We have tested additional compounds with similar properties, e.g., RO7015738, and have confirmed that GABA_A_-α5 PAMs reduce repetitive behaviors in these mouse models (Buettelmann et al., 2018). These results align well with recently published data showing that GABA_A_-α5 receptor deficiency causes autism-like behaviors in mice, including increased grooming and reduced social contacts (Zurek et al., 2016), and with SH-053-2′F-R-CH3, a GABA_A_-α5 PAM, which was recently shown to have positive effects on social and cognitive deficits in a rat model of autism (Souza et al., 2024). Our data are valuable in providing information on the RO required for *in vivo* effects on repetitive behavior. However, it should be noted that this effect may not directly translate to all individuals with ASD as it is a heterogeneous disorder, whereas these mouse models exhibit specific genetic or phenotypic deficits.

### 4.4 Anticonvulsant effects of alogabat

It has been proposed that the reduction of GABAergic inhibitory activity results in hyperexcitability of minicolumn circuits, explaining some of the symptomatology observed in some NDDs, including the high incidence of seizures and auditory–tactile hypersensitivity (Rubenstein and Merzenich, 2003; Frye et al., 2016). In the current study, we demonstrated that alogabat exhibits an anticonvulsant profile with activity comparable to the benzodiazepine drug class in acutely induced seizure models (electrical and chemical). Alogabat had a small effect on seizures in the MES test, whereas in the PTZ test, alogabat was shown to completely block seizures at calculated GABA_A_-α5 RO >70%. Since PTZ is a GABA_A_ antagonist, this supports the assessment of alogabat for anticonvulsant activity in disorders where there is dysfunction of the GABAergic system. Furthermore, in a separate EEG study, we observed that one out of eight rats had frequent spike-and-wave discharge (SWD) activity, which was alleviated following treatment with alogabat (see Supplementary Figures S3A–S3C; Methods in Supplementary Material). To better understand the temporal dynamics of SWD occurrence and the suppressing effect of alogabat, we performed a follow-up study on three additional rats that showed pronounced SWD. Both 3 and 10 mg/kg alogabat substantially reduced the number of SWDs within the first 3 h of recording, when the same rats showed increased SWD numbers in the vehicle control condition (see Supplementary Figures S3D–S3F), which corresponds to the calculated GABA_A_-α5 RO level of 30%–46% (see Supplementary Table S6). From a circuit perspective, the consensus is that such epileptic activity is generated by dysregulated thalamocortical circuitry, with abnormal interactions between reticular thalamic neurons and thalamic relay cells acting as the trigger and cortical circuits serving as the generator (Sitnikova et al., 2023). Considering that in rats, α5-subunit-containing GABA_A_ receptors are enriched in the cortex and hippocampus (Serwanski et al., 2006; Hu et al., 2019), mediating dendritic inhibition on pyramidal neurons (Ali and Thomson, 2008; Schulz et al., 2018), we hypothesize that alogabat could suppress SWD generation by enhancing the inhibition of cortical pyramidal neurons. However, more systematic work is required to confirm that alogabat can prevent SWD activity.

### 4.5 Assessment of non-specific behavioral effects

Non-selective GABA_A_ PAMs, such as diazepam and chlordiazepoxide, are effective anxiolytics in clinical use but have also been shown to impair cognition in rodents and humans. GABA_A_-α5 receptors are believed to play a major role in cognition since they are predominantly expressed in the hippocampus in rats, where they regulate NMDAR-dependent synaptic plasticity underlying learning and memory processes (Schulz et al., 2018; Fritschy et al., 1997; Schulz et al., 2019; Davenport et al., 2021). Moreover, it has been proposed that GABA_A_ PAMs may induce memory impairment by modulating hippocampal function (McNaughton and Morris, 1987). Therefore, alogabat was evaluated in several rodent tests of cognition to determine whether a selective GABA_A_-α5 PAM would induce cognitive impairment following acute administration in rats. There was no effect of alogabat up to 70% GABA_A_-α5 RO on cognition (visual–spatial memory, attention, context fear conditioning, spatial learning, and memory). Cognitive impairment was only observed at high doses (>70% RO) and supra-therapeutic levels of alogabat in associative (fear) learning (CFC) and spatial memory in the MWM in wildtype rats. The impairment at high doses is likely linked to the loss of selectivity toward GABA_A_ α1, α2, and α3 receptor subtypes (α1 RO was calculated as 19%–25% at these doses; see Supplementary Table S6). In addition to the loss of selectivity, we cannot exclude the possibility that these effects may be a consequence of GABA_A_-α5 receptor saturation, i.e., excessive α5 activity in wildtype subjects may be detrimental. The cognitive impairment observed in wildtype rats may translate to healthy human subjects; however, our focus is on the effect of α5 PAMs in disease conditions with impaired GABA_A_ signaling and impaired cognition, where our hypothesis is that an α5 PAM would have beneficial effects.

Alogabat had no effect on social approach/avoidance, the elevated plus maze, and the Vogel conflict test in rats at RO levels up to 80% (see Supplementary Figure S4 and Methods in Supplementary Material), suggesting that alogabat does not exhibit an anxiolytic-like effect in rodents. This might be explained by the low expression of GABA_A_-α5 in the amygdala, a key node controlling anxiety-like behaviors. Potential anxiolytic effects of alogabat in humans cannot be excluded as this receptor subtype is much more abundant in the human amygdala. Since benzodiazepines, such as diazepam, are frequently used to treat anxiety and seizures in NDDs, it was important to establish that alogabat at relevant therapeutic doses in mouse models did not worsen the sedative/motor-impairing effects of diazepam in the rotarod test. Alogabat did not worsen diazepam-induced motor impairment; on the contrary, in combination, it reduced the motor impairments, which is more likely due to the more localized and precise inhibitory effect, counterbalancing the broader suppression of neuronal activity by diazepam, or due to lower intrinsic activity at α1 receptors compared to diazepam. We have previously shown that a GABA_A_-α5 NAM, which binds at the benzodiazepine binding site of the receptor, does not decrease the anxiolytic activity of chlordiazepoxide (see Supplementary Figure S5); therefore, it is unlikely that alogabat will interfere with the anxiolytic effect of the classical benzodiazepines.

## 5 Conclusion

Multiple lines of evidence point to deficits in GABAergic signaling in epilepsy and NDDs, such as ASD and deletion AS, with a prominent role attributed to GABA_A_ receptors, including the GABA_A_-α5 receptor subtype. The current data for alogabat showed robust dose-dependent efficacy on repetitive behaviors in two disease-relevant mouse models and anti-seizure activity in the PTZ rat model at GABA_A_-α5 RO above 50%. Our *in vivo* occupancy measurements using the selective α5 tracer L-655,708 were highly valuable for understanding plasma and CSF exposures (as a surrogate of free brain concentration) and their relationship to *in vivo* effects. We observed that there was a 34-fold shift between the CSF concentration of alogabat in the *in vivo* occupancy study and that of alogabat *in vitro* binding data (Ki value). The receptor occupancy of alogabat correlates well with PD effects (*in vivo* effects) and is consistent across the different studies/species (see Supplementary Table S6). This finding highlights the importance of using PET tracers in drug development.

Our data suggest that GABA_A_-α5 PAMs may have potential benefits in individuals with autistic features and in conditions with clinical and nonclinical manifestations of cortical hyperexcitability and epilepsy. However, alogabat is unlikely to work across all NDDs and should be utilized as a precision medicine to target a specific subgroup of the NDD population with deficient GABA_A_ pathway signaling, or specifically GABA_A_-α5. Interestingly, recent data suggest that GABA_A_-α5 PAMs may also have therapeutic potential in schizophrenia, Alzheimer’s disease, and depression (Perez et al., 2023; Luscher et al., 2023; Koh et al., 2013). With regard to the current status of alogabat, good-laboratory practice (GLP) toxicology and safety pharmacology studies in rats and minipigs showed an overall benign safety profile, which supported its clinical investigations in phase-1 studies in healthy volunteers and phase-2 trials in ASD and deletion AS patients (NCT03507569, NCT03774576, NCT03847987, NCT04299464, NCT05630066, and EudraCT#2019-003524-20). The outcome of these studies, including PET, EEG, and effects on cognition, will be reported in the future.

## Data Availability

The original contributions presented in the study are included in the article/Supplementary Material, further inquiries can be directed to the corresponding author.

## Abbreviations

AS, Angelman syndrome; ASD, autism spectrum disorder; CFC, context fear conditioning; Cntnap2, contactin-associated protein-like 2; CPT, continuous performance test; 5-CSRT, 5-choice serial reaction time; EEG, electroencephalography; GABA, gamma-aminobutyric acid; GAD, glutamic acid decarboxylase; i.p., intraperitoneally; i.v., intravenously; LFP, local field potential; MES, maximum electrical stimulus; MWM, Morris water maze; NDDs, neurodevelopmental disorders; PAL, paired associates learning; PAM, positive allosteric modulator; phMRI, pharmacological magnetic resonance imaging; p.o, per os; PTZ, pentylenetetrazole; RO, receptor occupancy; PET, positron emission tomography.
