# Supplementary material for: Preclinical pharmacology of alogabat: a novel GABAA-α5 positive allosteric modulator targeting neurodevelopmental disorders with impaired GABAA signaling
Source: Front Pharmacol. 2025 Jul 21;16:1626078. doi: 10.3389/fphar.2025.1626078 (PMC12319775; doi:10.3389/fphar.2025.1626078)
Supplement: Supplementary file 1 [file Supplementaryfile1.docx]

Supplementary Material

# SUPPLEMENTARY IN VITRO METHODS

Alogabat was prepared as a 10 mM stock solution in DMSO for all in vitro experiments.

## GABA_A_ RECEPTOR RADIOLIGAND BINDING ASSAYS

***Plasmids and recombinant cell expression - Rat receptors:*** cDNAs encoding the rat GABA_A_ α1, α2, α3, α5, β2, β3 and γ2 subunits receptors were cloned into the pFastBac transfer vector (Invitrogen) using a modified Kozak sequence for optimal translational efficiency. The coding DNAs were introduced into the viral genome by homologous recombination and then expressed in SF9 insect cells. The baculovirus/SF9 system was selected for expression as it allows the infection of SF9 cells with recombinant viruses at different ratios (different MOI’s) to get the highest expression yields for a given receptor subtype.

To generate stable cell lines expressing different rat GABA_A_ receptor subtypes, cDNAs encoding the rat GABA_A_ α1, α2, α3 and α5 receptor subunits were subcloned into the pIRESpuro vector and the cDNAs encoding the rat GABA_A_ β2 and β3 and γ2 (short) receptor subunits were subcloned into the pIRESneo and pIREShygro vector, respectively (Clontech, Mountain View, CA). Stable cell lines expressing rat GABA_A_ α1β2γ2, α2β3γ2, α3β3γ2and α5β3γ2 receptor subtypes were derived by transfection of pIRES plasmids containing the desired subunit cDNAs (α, β, γ, 1:1:2 ratio) into HEK 293 cells, using the lipofectamine 2000 kit according to the manufacturer’s instructions (Invitrogen, Carlsbad, CA, USA). The cells were grown in minimal essential medium (Invitrogen) supplemented with 10% fetal calf serum (Invitrogen), 20 mM HEPES (Invitrogen) and 100 U/ml penicillin/ 100 μg/ml streptomycin (Invitrogen). The culture medium for the stable cell lines contained, in addition, 0.3 μg/ml puromycin (Clontech, Mountain View, CA, USA), 300 μg/ml hygromycin B (Roche Diagnostics, Mannheim, Germany) and 200 μg/ml G418 (Invitrogen). For electrophysiological experiments, the cells were plated onto glass coverslips coated with poly-D-lysin (Sigma-Aldrich, St. Louis, MO, USA).

***Plasmids and recombinant cell expression - Human receptors:*** The complementary DNAs (cDNA) encoding different human GABA_A_ receptor subunits (α1-6, β2‑3, γ2) were subcloned into the polylinker of the pcDNA3.1 vector (Invitrogene USA) by standard techniques for transiently transfect human embryonic kidney (HEK)293-EBNA cells or for in vitro RNA synthesis.

HEK293-EBNA cells adapted to grow in suspension were transiently transfected with the plasmids containing the desired GABA_A_ receptor subunit cDNAs at α, β, γ, 1:1:2 ratio using XtremeGENEQ2 (Cat. No. 03045595001, Roche Applied Science, RAS, Rotkreuz, Switzerland) as previously described (for methods refer to (Malherbe et al., 2008). GABA_A_ α1β3γ2, α2β3γ2, α3β3γ2, α4β3γ2, α5β3γ2 and α6β3γ2 receptor subtypes were expressed in this system. 48 h post-transfection, the cells were harvested and washed three times with cold PBS and frozen at –80°C.

***Membrane preparation:*** The pellet from cells expressing the different GABA_A_ receptor subtypes, either SF9 or HEK293 cells, was suspended in cold 15 mM Tris-HCl, 120 mM NaCl, 100 mM KCl, 25 mM CaCl2, 25 mM MgCl2 (pH=7.4) and homogenized with a polytron (Kinematica AG, Basel, Switzerland) for 10 s at 18,000 rpm. After centrifugation at 50,000 x g for 60 min at 4°C, the supernatant was removed, and the pellet was polytronized for 10 s at 18,000 rpm. This pellet was further resuspended in a smaller volume of ice cold 50 mM Tris-HCl buffer containing 0.1 mM EDTA (pH 7.4). The protein content was measured using the Bradford method (Biorad Laboratories GmbH, Munchen, Germany) with Gamma globulin as the standard. The membrane homogenate was frozen at –80°C before use.

[^3^H]flumazenil binding assays for GABA_A_ receptors containing α1, α2, α3, and α5

Receptor binding experiments were performed as previously described (Ballard et al., 2009). After thawing, the membrane homogenates were resuspended and polytronized in 15 mM Tris-HCl, 120 mM NaCl, 5 mM KCl, 1.25 mM CaCl2, 1.25 mM MgCl2 binding buffer at pH 7.4 to a final assay concentration of up to 100 μg protein depending on the membrane prep receptor expression / well. Saturation isotherms were determined by addition of twelve radioligand concentrations to these membranes (in a total volume of 200 μl) for 1 h at 4°C for [^3^H]flumazenil. At the end of the incubation, membranes were filtered onto unifilter (96-well white microplate with bonded GF/C filters) and were preincubated 20-30 min in cold assay buffer) with a Filtermate 196 harvester (Packard BioScience) and washed 3 times with cold 50 mM Tris-HCl, pH 7.4 buffer. Nonspecific binding was measured in the presence of 10 μM Diazepam.

For inhibition experiments, membranes were incubated with 1 nM of [^3^H]flumazenil (or adjusted to the specific receptor subtype Kd) and ten concentrations of alogabat. IC_50_ values were derived from the inhibition curve and Ki values were calculated according to the following equation Ki = IC_50_/(1+[L]/Kd) where [L] is the concentration of radioligand and Kd is its dissociation constant at the receptor, derived from the saturation isotherm. The radioactivity on the filter was counted (3 min) on a Topcount microplate scintillation counter (Packard).

[^3^H]RO0154513 binding assays for GABA_A_ receptors containing α4 and α6

The affinity of alogabat for GABA_A_ receptors containing α4 and α6 subunits was measured using [^3^H]RO0154513 which shows high affinity for these receptor subtypes.

After thawing, the membrane homogenates were resuspended and polytronized in 15 mM Tris-HCl, 120 mM NaCl, 100 mM KCl, 25 mM CaCl2, 25 mM MgCl2 binding buffer at pH 7.4 to a final assay concentration of up to 100 μg protein depending on the membrane prep receptor expression / well. Saturation isotherms were determined by addition of twelve radioligand concentrations to these membranes (in a total volume of 200 μl) for 60 min at 4°C for [^3^H]RO0154513. At the end of the incubation, membranes were filtered onto unifilter (96-well white microplate with bonded GF/C filters) and were preincubated 20 min in cold assay buffer) with a Filtermate 196 harvester (Packard BioScience) and washed 3 times with cold 50 mM Tris-HCl, pH 7.4 buffer. Nonspecific binding was measured in the presence of 10 μM RO0154513. For inhibition experiments, membranes were incubated with 1 nM of [^3^H]RO0154513 (or adjusted to the receptor subtype Kd) and ten concentrations of alogabat. IC50 values were derived from the inhibition curve and Ki values were calculated according to the following equation Ki = IC_50_/(1+[L]/Kd) where [L] is the concentration of radioligand and Kd is its dissociation constant at the receptor, derived from the saturation isotherm. The radioactivity on the filter was counted (3 min) on a Topcount microplate scintillation counter (Packard).

Data calculation

The CPM value for each duplicate of a concentration of competing compound was averaged (y1), then the % specific binding was calculated according to the equation (((y1 – nonspecific)/(total binding-nonspecific))x100). Graphs were plotted with the % specific binding using XLfit, a curve fitting program that iteratively plots the data using Levenburg Marquardt algorithm. The single site competition analysis equation used was y = A + ((B-A)/(1+((x/C)D))), where y is the % specific binding, A is the minimum y, B is the maximum y, C is the IC_50_, x is the log10 of the concentration of the competing compound and D is the slope of the curve (the Hill Coefficient). From these curves the IC50 (inhibition concentration at which 50% specific binding of the radioligand was displaced) and Hill coefficient were determined. The affinity constant (Ki) was calculated using the Cheng-Prusoff equation Ki = (IC_50_/1+([L]/Kd), where [L] is the concentration of radioligand and Kd is the affinity constant of the radioligand. Saturation experiments were analyzed by XLfit using the Michaelis-Menten Equation derived from the equation of a bimolecular reaction and the law of mass action, B = (B_max_ * [F])/(Kd + [F]), where B is the amount of ligand bound at equilibrium, B_max_ is the maximum number of binding sites, [F] is the concentration of free ligand and Kd is the ligand dissociation constant. For inhibition experiments, membranes were incubated with 2 nM of [^3^H]x and ten concentrations of ROx or other inhibitory compounds. IC_50_ values were derived from the inhibition curve and Ki values were calculated according to the following equation Ki = IC_50_/(1+[L]/Kd), where [L] is the concentration of radioligand and Kd is its dissociation constant at the receptor, derived from the saturation isotherm with quenching correction after addition of 45 μl of microscint 40 and shaking for 120 min.

## GABA_A_ RECEPTOR ELECTROPHYSIOLOGICAL STUDIES

Whole cell patch-clamp of HEK293 cells containing rat GABA_A_ receptors

Experiments were performed with HEK293 cells expressing the α1β2γ2, α2β3γ2, α3β3γ2, and α5β3γ2 GABA_A_ receptor subtypes. Cells plated on glass coverslips were transferred to a Perspex chamber on the stage of Nikon Diaphot inverted microscope and continuously perfused with a solution containing (in mM) NaCl 150, KCl 4, CaCl_2_ 1.2, MgCl_2_ 1, HEPES 10, pH 7.2 adjusted to 7.4 with NaOH and osmolarity adjusted to 340 mOsm with sucrose. Patch pipettes were pulled with an approximate tip diameter of 2 μm and a resistance of 2 MΩ with borosilicate glass (Clark Electromedical Instruments, Reading, UK) and filled with a solution containing (in mM) CsCl 140, HEPES 10, ethylene glycol tetraacetic acid 11, MgCl_2_ 1, CaCl_2_ 1, and Mg-ATP 4, pH adjusted to 7.2 with CsOH and osmolarity adjusted to 340 mOsm with sucrose. Cells were recorded in the patch-clamp whole cell mode using an Multiclamp 700B amplifier (Molecular Devices, Sunnyvale, CA, USA). GABA in the presence or absence of alogabat was applied to the cell for 2 s in 1 min intervals using a multibarrelled microapplicator pipette controlled by a stepping motor (Bio-Logic SAS, Claix, France). For each experiment, at least three GABA control applications were generated and only cells showing stable GABA responses were selected for the compound testing. Prior to application of a GABA–compound mixture, the same concentration of alogabat alone was applied by bath perfusion for 1 min. During the experiment, all bath solutions contained 0.1% DMSO, which by itself was without detectable effect on the GABA responses.

***Data analysis:*** The pClamp data acquisition program set (Molecular Devices) and GraphPad Prism (GraphPad Software LLC, San Diego, USA) were used for data acquisition and analysis. To estimate the modulation by alogabat of the GABA-induced current, the maximum current amplitudes measured in the presence of GABA and alogabat were normalized to the maximum current amplitude measured in the presence of GABA alone for each cell. The % modulation values were plotted as a function of the concentration and fitted using the equation:

% modulation(x) = % Maximum modulation/[1+(x/EC_50_)^n^],

where % Maximum modulation is the maximum modulation by alogabat of the GABA-induced current, x the concentration of alogabat, EC_50_ is the half-maximum effective alogabat concentration, and n the Hill slope.

Voltage clamp in *Xenopus* Oocytes containing human GABA_A_ receptors

This set of experiments was performed in collaboration with HiQScreen Sàrl., Genève Switzerland.

***Cloning and preparation of RNA for microinjection into Xenopus oocytes:*** The cDNAs encoding different human GABA_A_ receptor subunits were subcloned into the polylinker of the pcDNA3.1 vector (Invitrogene, USA) by standard techniques. The constructs were linearized at the 3’ end of the corresponding receptor subunit cDNA with the appropriate restriction enzyme keeping the polymerase promoter site upstream of the sequence to be transcribed (see **Table 1**). Capped and poly(A) tailed cRNA transcripts were synthesized from linearized plasmids encoding the desired protein by using the mMessage mMachine T7 ultra kit (Ambion) according to the recommendation of the manufacturer. The synthesized RNA was purified by using the MEGAclear (Ambion) spin columns. cRNA concentrations were calculated with the Nanodrop 8000 and visualized on 1.5% agarose gels. The RNAs are stored at – 80 °C.

**Table 1.** Restriction enzyme sites used to linearize the different GABA_A_ receptor subunit cDNAs cloned into the pcDNA3.1 vectors

| **cDNA** | **Vector** | **Restriction site** |
| --- | --- | --- |
| GABRA1_HUMAN | pcDNA 3.1(-) | Hind III |
| GABRA2_HUMAN | pcDNA 3.1(+) | Xba I |
| GABRA3_HUMAN | pcDNA 3.1(-) | Hind III |
| GABRA5_HUMAN | pcDNA 3.1(-) | Hind III |
| GABRB2_HUMAN | pcDNA 3.1(+) | Xba I |
| GABRB3_HUMAN | pcDNA 3.1(-) | Not I |
| GABRG2_HUMAN | pcDNA 3.1(+) | Xba I |

Currents evoked by GABA were recorded using the standard two electrode voltage-clamp configuration (TEVC) with an automated voltage-clamp screening system (HiClamp, Multichannelsystems, Reutlingen Germany). *Xenopus* oocytes were prepared and injected using standard procedures. Briefly, ovaries were harvested from *Xenopus Laevis* females that had been deeply anesthetized and pithed following the animal rights rule from the Geneva canton. A small piece of ovary was isolated for immediate preparation while the remaining part was placed at 4 ^o^C in a sterile Barth solution containing in mM NaCl 88, KCl 1, NaHCO_3_ 2.4, HEPES 10, MgSO_4_.7H_2_O 0.82, Ca(NO_3_)_2_.4H_2_O 0.33, CaCl_2_.6H_2_O 0.41, at pH 7.4, and supplemented with 20 μg/ml kanamycine, 100 unit/ml penicillin and 100 μg/ml streptomycin. On the second day following dissociation, oocytes were injected with 5 ng of mRNA encoding for the various GABA receptor subunit combinations per oocyte using an automated injector (Roboinject, Multichannelsystems). All recordings were performed at 18 °C and oocytes superfused with OR2 medium containing in mM: NaCl 82.5, KCl 2.5, HEPES 5, CaCl_2_.2H_2_O 2.5, pH 7.4. Oocytes were held at –80 mV.

***Compound application:*** 1–2 min after impaling an oocyte in a well of a 96-well plate containing OR2 and switching to voltage-clamp mode, the oocyte was transferred to a well containing OR2 with GABA for 100 s to record a control response. The oocyte was then washed with OR2 for 160 s and transferred again to a well containing OR2 with GABA. To test the effect of alogabat on the GABA response, the oocyte was transferred 40 s after the onset of the GABA response to a well containing GABA in the presence of alogabat for 60 s. Each oocyte was used for testing only one concentration of alogabat.

***Data analysis:*** Data were filtered at 10 Hz, recorded at 100 Hz and analyzed using proprietary data acquisition using Matlab (Mathworks Inc., Portola Valley, California, U.S.A.). The modulation of the GABA-induced current by alogabat was estimated using the same procedure as that described above with HEK293 cells.

## RAT HIPPOCAMPAL SLICE ELECTROPHYSIOLOGICAL STUDIES

***Slice preparation:*** 1–3 months old Wistar rats (Charles River, Germany) were used in the population spike recording (1 rat/3 slices) and paired-pulse inhibition (0.3 μM: 1 rat/3 slices; 1 μM: 2 rats/6 slices) experiments. Animals were anaesthetized in a 2.5% isoflurane / 96.5% oxygen mixture and sacrificed. The hippocampi were dissected, 400-μm slices were transversely cut with a tissue chopper (Sorvall, Newton, USA) and placed in a perfusion chamber for later used.

***Population spike and paired-pulse inhibition recordings:*** Rat hippocampal slices were maintained in a submerged chamber and perfused at room temperature in a solution containing (in mM): NaCl 124, KCl 5, MgSO4 2, CaCl2 2, KH2PO4 1.25, NaHCO3 25, D-glucose 11 gasses with 95% O2/5% CO2 (pH 7.4, 307 mOsm). Population spikes (PS) were recorded from the CA1 region of the hippocampus. A glass micropipette (1–3 MΩ) containing 2 M NaCl was positioned in the stratum pyramidale and insulated bipolar platinum iridium electrodes were positioned in the Schaffer collaterals for orthodromic stimulation (0.1 ms, 60-90 μA). The stimulus intensity that induced a PS near to the maximum amplitude was used as the test stimulus. PS were amplified with a Cyberamp 380 amplifier (Molecular Devices), filtered at 2.4 KHz and digitized at 20 KHz with a Digidata 1322 acquisition board (Molecular Devices) for subsequent storage on a personal computer (Compaq Deskpro EN).

***Data analysis:*** To obtain concentration-response relationships, PS were evoked at 30 s intervals and increasing concentrations of isoguvacine were applied to the slice until a maximum inhibition of the PS was reached. For each concentration of isoguvacine, the PS amplitude obtained from four averaged responses was measured and isoguvacine was then removed from the salt solution. After a full recovery of the PS, alogabat was preapplied to the slice for 15 min and another isoguvacine concentration response curve was generated in the presence of the compound.

PS amplitudes were fitted with the nonlinear least-squares fitting routine of XLFit using the equation:

PS(x) = PS_max_ / [1 + (x/IC_50_) n]

where PS is the measured PS amplitude, PS_max_ the maximum PS amplitude, x the concentration of isoguvacine, IC_50_ the half-maximum effective concentration and n the Hill slope.

# SUPPLEMENTARY IN VIVO METHODS

## RECEPTOR OCCUPANCY STUDIES

Animals and Housing

Male Wistar rats (~160 g, Charles River, Germany) and Cntnap2-/- mice (18-22 g, bred at Roche Basel) were group-housed in separate holding rooms at controlled temperature, humidity and 12 h light/dark cycle and had ad libitum access to food and water.

Treatment Protocol

**Male Wistar rats** (n=26) were randomly assigned to treatment with either vehicle or 0.3, 1, 3, 10, 30 or 100 mg/kg alogabat intraperitoneally (i.p.; n=3/group) and 15 min later received 0.1 mCi/kg [^3^H]RO0154513 (equivalent to 0.8 µg/kg) intravenously (i.v.) using a similar experimental approach described previously (Ballard et al., 2009; Hipp et al., 2021). For determination of non-specific binding of [^3^H]RO0154513, two rats received the GABA_A_-α5 blocker L-655,708 at 10 mg/kg i.p. (Atack, Alder, Cook, Smith, & McKernan, 2005). Rats were sacrificed by decapitation (under isoflurane anesthesia) 15 min after administration of the radioligand and brains and blood were collected for analysis of specific binding and drug concentration.

**Cntnap2-/- mice** (n=16) were randomly assigned to treatment with either vehicle or 30, 60 or 100 mg/kg alogabat i.p. (n=4/group). Mice were sacrificed 30 min later by decapitation (under isoflurane anesthesia) and brains and blood were collected for analysis of specific binding and drug concentration. Receptor occupancy was assessed in brain sections in vitro with the highly selective GABA_A_-α5 radioligand, [^3^H]L-655,708.

Brains and blood were collected from each animal. Blood was collected in Heparin-Lithium tubes (Milian, Basel, Switzerland) and plasma submitted to the Roche analytical laboratories for the determination of plasma levels of alogabat. Brains were rapidly removed, divided in two halves along their sagittal axis and frozen in dry ice. Half brain was submitted to the Roche analytical laboratories for the determination of the brain concentrations of alogabat. The other half was placed in a cryostat and sagittal sections (10 μm thickness, 3-4 sections per brain) were mounted on Histobond glass slides (Marienfeld Laboratories Glassware, Germany).

In vivo occupancy in Wistar rats

The brain sections were dried at room temperature and exposed, together with tritium microscales, to tritium sensitive imaging plates (BAS-TR2025) for five days. The imaging plates were scanned in a Fujifilm BAS-5000 high resolution phosphor imager and the amount of [^3^H]RO0154513 bound to the brain regions of interest was quantified with an MCID M2 image analysis system (Imaging Research Inc., St. Catherines, Ontario, Canada) and expressed as fmol of [^3^H]RO0154513/mg of protein.

Ex vivo occupancy in Cntnap2-/- mice

The brain sections were incubated with 2 nM [^3^H]L655,708 for 5 min at 4°C and washed twice for 1 min in ice-cold buffer, followed by two dips in ice-cold water. Sections were dried at room temperature and exposed, together with tritium microscales, to tritium-sensitive imaging plates (BAS-TR2025) for five days.

Data analysis

**For the Wistar rat in vivo occupancy study**: non-specific binding (NSB) was determined by treating one group of animals with the established GABA_A_-α5 receptor blocker L-655,708 (10 mg/kg i.p.) 30 min prior to radioligand injection. Specific binding (SB) of [^3^H]RO0154513 in the regions-of-interest (ROI) was calculated by subtracting NSB from totally bound [3H]RO0154513 (TB) according to the equation: SB = TB – NSB.

**For Wistar rat and Cntnap2-/- mouse studies**: receptor occupancy produced by alogabat was calculated according to the following equation:

% receptor occupancy = (1-(SB_alogabat_/SB_vehicle_)) x 100

where “SB_alogabat_” is the SB measured in the ROI of rats pre-treated with alogabat and “SB_vehicle_” is the SB measured in the ROI of rats pre-treated with vehicle.

Data were analyzed by XLfit (equation 350). The following equation was used to estimate O_max_ and EC_50_:

Occupancy = (O_max_ * [x])/(EC_50_ + [x])

where O_max_ is the theoretical maximal occupancy, [x] is the plasma or the brain concentration of alogabat and EC_50_ is the plasma or brain concentration of alogabat that produces 50% receptor occupancy.

## PHARMACOLOGICAL MAGNETIC RESONANCE IMAGING IN RATS

Animals and Housing

Naïve male Fischer rats (n=32, ~250 g, Charles River, Germany) were group-housed in holding rooms at controlled temperature, humidity and 12 h light/dark cycle and had ad libitum access to food and water.

Animal preparation

For magnetic resonance imaging (MRI), rats were randomly assigned to treatment with either a 3, 10 or 30 mg/kg dose of alogabat intraperitoneally (i.p.), or an equivalent volume of vehicle as negative control (n=8/dose). After 15 minutes, rats were initially anaesthetized using isoflurane (4%) in carrier gas composed of oxygen and air (1:5) supplied to the spontaneously breathing animals in an inhalation box. Upon induction of anesthesia a s.c. bolus injection of 0.2 mg/kg (1 ml/kg) medetomidine prepared from 1:5 diluted Dormilan® was given and the animal was transferred onto a rat cradle for inserting a s.c catheter and starting continuous infusion of 1:10 diluted Dormilan® (medetomidine) at a dose of 0.1 mg/kg/h (1 ml/kg/h). The head was immobilized in a stereotaxic frame. Respiratory rate, body temperature, and O_2_ and CO_2_ levels in the inhaled and exhaled air were continuously monitored on a PowerLab data acquisition system (ADInstruments, Spechbach, Germany). Body temperature was maintained at 37 °C with a feedback-regulated electric heating blanket. In total, each animal was subjected to medetomidine for approximately 40 min. Immediately after the last imaging assessment, rats were sacrificed by decapitation under anesthesia and plasma samples were collected.

Magnetic Resonance Imaging

Data was acquired on a 4.7 T/40 cm Bruker Biospec horizontal-bore small-animal scanner (Bruker BioSpin, Ettlingen, Germany), equipped with a 72 mm bird-cage resonator for excitation and an actively decoupled quadrature surface receiver coil (Rapid Biomedical, Rimpar, Germany) for head imaging. On scout images, the most rostral extension of the corpus callosum was used as a landmark for selecting eight coronal image planes at –10.0, –7.8, –5.3, –2.9, –1.6, –0.3, +1.0 and +2.3 mm from bregma (Paxinos & Watson, 1986). All subsequent images were acquired from these planes, with a field of view of 4 cm × 4 cm and a slice thickness of 1 mm. The first imaging volume was a set of rapid acquisition with relaxation enhancement (RARE) T2-weighted anatomical images (TR/TE_eff_  = 1800ms/39ms, RARE factor = 8, matrix = 256 × 256) (Hennig, Nauerth, & Friedburg, 1986). Next, a T1–weighted image series required to quantitatively calibrate perfusion readouts was obtained using an inversion-recovery snapshot FLASH sequence with 8 inversion times (TR/TE = 3400ms/1.4ms, matrix = 128 × 64) (Haase, Frahm, Matthaei, Hanicke, & Merboldt, 2011). Finally, perfusion weighted images were acquired using continuous arterial spin labelling (CASL) (Williams, Detre, Leigh, & Koretsky, 1992) with single-slice centered RARE readout (TR/TE = 3750ms/5.7ms, RARE-factor = 32, matrix = 128 × 64, 2.5s labelling pulse, 0.4s post-labelling delay). Three consecutive volumes of CASL perfusion images were acquired over an acquisition time of 4 minutes per volume.

Data Analysis

Images were processed and analyzed using in-house developed software written in IDL (RSI, Boulder, CO, USA) and MATLAB (The MathWorks Inc., Natick, MA, USA). The anatomical volume of each individual animal was co-registered to an in-house established rat-brain template using the open-source software SPM5 (Wellcome Trust Centre for Neuroimaging, London, UK). Spatial normalization comprised a 12-parameter affine as well as a nonlinear transform, which were then applied identically to all functional images of the same subject. The template was aligned and annotated with an in-house generated digital atlas that delineated 45 regions of interest (ROIs) adapted from the Paxinos & Watson rat-brain atlas (Paxinos & Watson, 1986). T1 maps were calculated on a voxel-wise basis by fitting a 3-parameter exponential to the image intensities across the 8 inversion times (Deichmann, Hahn, & Haase, 1999). These T1 maps were then combined with the related CASL images to obtain quantitative absolute perfusion maps as described elsewhere (Alsop & Detre, 1996; Bruns, Kunnecke, Risterucci, Moreau, & von Kienlin, 2009). In order to account for possible systemic changes affecting global brain perfusion, and to eliminate part of the inter-individual variability, perfusion maps of each individual were normalized slice-wise to the brain-mean value, which was set to 100%. Perfusion values were averaged ROI-wise (pooling hemispheres for bilateral ROIs) across the three consecutive acquisitions.

Statistics

Analysis was performed with JMP (SAS Institute Inc, Cary, USA) and MATLAB (The MathWorks Inc., Natick, MA, USA). Global (whole-brain) absolute-perfusion values from the vehicle and the 3 dose groups were tested for a linear trend across the 4 doses within the framework of a 1-way ANOVA. The same analysis was applied ROI-wise to the normalized-perfusion values. Multiple testing across the single ROIs was accounted for by controlling the false discovery rate (FDR) at 10 % using the Benjamini-Hochberg approach. To also obtain an overall activation-strength metric, normalized-perfusion values of each dose group were compared ROI-wise to those of the vehicle group using Welch’s t-test without multiple-testing correction. The number of significantly modulated ROIs at each dose was then taken as a measure of “pattern strength”. The mean and the upper 95% confidence limit of the corresponding chance levels were estimated via random group-label permutations (100,000 runs per group). Significance levels of actual pattern strengths were determined from the distributions obtained from the permutation procedure.

## EEG IN RATS

Animals and Housing

EEG studies in male Wistar rats (n=17, Envigo, USA) were conducted in accordance with the Guide for the Care and Use of Laboratory Animals (National Research Council 2011) after approval by the Institutional Animal Care and Use Committee by Brains Online (CA, USA). All rats were group-housed in holding rooms at controlled temperature, humidity and 12 h light/dark cycle and had ad libitum access to food and water.

Surgery

Rats were anesthetized using isoflurane (2%, 800 mL/min O_2_). Bupivacain/ epinephrine was used for local analgesia, Finadyne or carprophen for peri-/post-operative analgesia, and amoxicillin for antibiotics. The animals were placed in a stereotaxic frame (Kopf instruments, USA). A subcutaneous pocket was made close to the dorsal flank. Transmitter (F50-EEE, Data Sciences International, Physio Tel F50-EEE Small Animal CNS Telemetry, 3 bipolar channels, Biopotential lead, outer diameter 0.3mm) was inserted into the pocket. The transmitter had 3 bipolar channels:

- Channel 1 (EEG): Two screws in the left hemisphere (Frontal: 11 mm anterior, 2.5 mm lateral; Central: 3 mm anterior, 3.5 mm lateral to lambda; follow (Visser et al., 2003), channels Fl and Cl).
- Channel 2 (LFP): A single wire electrode targeting CA3 region of the right hippocampus (1st lead of bipolar channel) using Watson and Paxinos coordinates and referenced to two connected screws placed above the cerebellum (2nd lead of bipolar channel). Brains were removed after the end of the drug challenge phase for histological examination to determine placement of depth electrode.
- Channel 3 (EMG): Placed in the neck muscle for recording EMG to aid sleep staging.

Indwelling cannulas were implanted in the right jugular vein for drug administration and in the right femoral artery for the serial collection of blood samples. After surgery, animals were kept individually in cages, with food and water ad libitum. Animals received a 5-day regime of antibiotics, and pain management as needed. Experiments started two weeks after surgery.

Experimental setup

For the first two experimental days (i.e., days with drug administrations), animals were randomly assigned to vehicle and then alogabat or vice versa. Then, if the setup allowed, diazepam (the positive control) was measured (another compound was measured following diazepam, but these results are not reported here). Between experimental days animals recovered for one week. On an experimental day, compounds were administered 1.5 hours after the onset of the dark cycle (i.e., dark cycle starting at 7:00 am and compound administered at 8:30 am) as a continuous zero order infusion for 5 min. EEG, LFP and EMG data were sampled at a rate of 500 Hz (samples/s) in freely moving rats. Data were acquired in raw data format, without any filters applied. Along with the EEG locomotion information was recorded by means of an accelerometer within the transmitter. EEG and PK data were acquired in 17 animals. Overall, 35 recordings were performed (1 to 4 recordings per animal). Some recordings were discarded due to large EEG/LFP artefacts, resulting in a total of 28 recordings with usable EEG data for quantitative analyses.

EEG data processing

Sleep staging (awake, NREM sleep or REM sleep) and artifact labeling was performed manually: each 10 s section was rated based on EEG, locomotion signal, EMG, and where necessary, video. Sleep staging started 1 h before dosing and ended at least 11 hours after dosing depending on the PK of the compound. Artefacted sections were located with a combined automatic and visual detection method and discarded from subsequent analyses.

Power spectral density was estimated using Morlet wavelet transform, with 0.6 octave frequency resolution (f/σf = 5.74) and 5∙σt length, with 50% overlap between windows, for logarithmically spaced frequencies ranging from 2 to 128 Hz (f = 2^(1:0.1:7) Hz) (https://roche.github.io/neuro-meeglet/) (Bomatter, Paillard, Garces, Hipp, & Engemann, 2024; Hipp, Hawellek, Corbetta, Siegel, & Engel, 2012).

Artifact free (i.e., data not labeled to contain artifacts) EEG data in the awake state was used for the analyses reported here. We first investigated effects on power over predefined theta and beta power ranges, defined a priori as 6–10 Hz and 20–30 Hz respectively based on previous studies with GABA_A_ drugs (van Lier, Drinkenburg, van Eeten, & Coenen, 2004; Visser et al., 2003). Then, we evaluated frequency-specific effects across the 2 to 128Hz range.

Statistical analysis

We used linear mixed effects models (LMMs). LMMs can account for incomplete datasets as is the case for this study. Specific contrasts were derived tested using t-tests within the model using Satterthwaite approximation for degrees of freedom.

We derived models for each compound individually: Y ~ COMPOUND + (1|ANIMAL)

The significance of differences in the peak frequency was assessed using random permutation tests (10,000).

## BEHAVIORAL ASSESSMENTS

Animals and Housing

All animals were group-housed by strain in holding rooms at controlled temperature (20–22°C), humidity (55-65%) and 12 h light/dark cycle (lights on at 06:00 h). All animals had ad libitum access to food and water, except those used in the operant conditioning tasks (food earned in test plus 12-15g/rat/day in the home cage) and in the Vogel conflict test (water restriction, see details below).

COGNITION TESTS

5-choice serial reaction time task

The 5-choice serial reaction time (5-CSRT) task training procedure was identical to that described in (Higgins, Ballard, Enderlin, Haman, & Kemp, 2005). The operant conditioning chambers (24 x 30.5 x 21 cm) were obtained from Med Associates Inc. (VT, USA) and were enclosed in individual sound attenuating boxes with a ventilation fan. The protocols were run by K-Limbic software from Conclusive Solutions (Harlow, UK). On one wall of the chamber was located a pellet receptacle equipped with a stimulus light and infra-red detector for head entries. On the opposite wall was located an array of 5 holes, each equipped with a cue light and infra-red head entry detector. Eleven food-deprived Lister Hooded (LH) rats (~400g, males, Charles River, Germany) were previously trained to nose-poke into the magazine tray to initiate the first trial which consisted of an inter-trial interval (ITI) of 5 s followed by the random illumination of one of the lights in the 5 holes for a fixed interval of 0.5 s (stimulus duration, SD). If a nose-poke was registered in the illuminated hole before the end of either the SD, or a fixed interval after this period of 5 s a food pellet was dispensed and a correct trial registered. An incorrect nose-poke (incorrect trial) or failure to respond within the allotted time (missed trial/omission) resulted in a time out period during which the house light was extinguished for 5 s. Premature responses were recorded as the number of nose-pokes into one of the five holes during the ITI. Perseverative responses were registered as the number of nose-pokes into one of the five holes after a correct trial was registered. Training continued until subjects had achieved consistent performance above a threshold of 75% correct ([correct/(correct + incorrect)] x 100) and <20% omissions for at least a 2-week period. Once animals have learned the task there is no memory component necessary for accurate performance, rather the animals must make a rapid (typically 0.5–0.6 s) response to a brief (0.5 s) flash of light presented unpredictably in 1 of 5 locations, i.e., 5-choice. This is a test of attention, independent of hippocampal function.

Touchscreen tasks: Habituation and Pre-training

The touchscreen chambers (32 x 25 x 25 cm) were obtained from Med Associates Inc. (VT, USA) and were enclosed in individual sound attenuating boxes with a ventilation fan. The protocols were run by K-Limbic software from Conclusive Solutions (Harlow, UK). Rats were tested in an operant chamber equipped with a touchscreen on one side and a pellet receptacle containing a light and an infra-red nose-poke detector on the opposite side. A house light and tone generator (2.9 KHz, 65 Db) were also present. The touchscreen was surrounded horizontally and vertically by infra-red beams, so that when the rat touched the screen the beams were broken and the location of the touch could be determined. Below the touchscreen a counter-weighted metal shelf was positioned to slow the rat’s approach and aid attendance to the response areas.

Each rat was first habituated to a touchscreen chamber for 15–20 min with no program running. Food pellets (45-mg Noyes formula ‘P’) were placed in the receptacle to encourage exploration. In the first pre-training stage, a white square (5 x 5 cm) was displayed indefinitely, or until the rat nose poked the square, at which point the animal received a single food pellet. Sessions terminated after 60 min or 100 pellets received. Sessions were continued until rats completed all trials during 2 consecutive days.

For the final stage of pre-training, rats were required to initiate the first trial. The house light was turned on and illumination of the pellet receptacle signaled the beginning of the trial. The rat initiated the trial by nose-poking the pellet receptacle. Subsequently, the pellet receptacle light was extinguished, and a white square appeared in the center zone. Responding on the white square triggered presentation of a tone, removal of the stimulus from the screen, turning on the pellet receptacle light and delivery of a single food pellet. Sessions terminated after 40 min or once 50 trials had been completed. Animals were trained until they achieved 50 correct trials for at least 2 consecutive days. Typically, 1–2 weeks of pre-training was required for animals to reach this criterion.

Continuous Performance Test

Following habituation and pretraining sessions, eleven male LH rats (~400g, Charles River, Germany) were then trained to asymptotic performance in the continuous performance test (CPT) using the procedure detailed below.

A black Perspex mask with one central window (10 cm x 10 cm) was positioned in front of the touchscreen giving one central response area. Only one stimulus (image) was presented at a time in a fixed, central position on the touchscreen. At the start of each session, the pellet receptacle was illuminated, a brief tone emitted, and a pellet delivered, so that the rats initiated the first trial by nose-poking in the pellet receptacle.

The first stage of CPT training consisted of the presentation of a stimulus (vertical or horizontal stripes) that remained the designated S+ stimulus throughout training (**Figure 1A**). Once the rats had nose poked the stimulus, they received a food reward (ingestion delay was 8 s). The stimulus was presented on the touchscreen for a fixed amount of time (i.e., the stimulus duration [SD]), which at this stage was 5 s, after which there was a short inter-stimulus interval (ISI) of 2 s and then the next stimulus was shown. The subjects had no control over the pace of the stimuli; thus, if no response was made, subsequent stimuli were presented at a fixed pace with an ISI of 2 s. The rats progressed onto the next stage once they had reached 100 correct responses within 60 min.

The second stage of training involved the addition of one S- stimulus (e.g., diagonal stripes; **Figure 1A**). Here there were four different possible outcomes (**Figure 1B**): a correct response i.e., hit (nose poke to the S+ stimulus), an incorrect response i.e., false alarm (nose poke to the S- stimulus), a missed trial (no response to the S+ stimulus) and a correct rejection (no response to the S- stimulus). Thus, the rats were trained to respond only to the S+, and to inhibit a response to the S-. During this stage the SD was 2 s and the ISI was variable 2 ± 1 s and the session length was reduced from 60 min to 40 min. The training data were then used to calculate the ‘Hit Rate’, which is the rate at which the rats make a correct response and the ‘False Alarm Rate’, which is the rate at which the rats make an incorrect response. These were calculated as follows:

| Hit Rate | = | Number of correct trials |
| --- | --- | --- |
|  |  | Number of correct trials + Number of missed trials |

| False Alarm Rate | = | Number of incorrect trials |
| --- | --- | --- |
|  |  | Number of incorrect trials + Number of correct rejections |

A high Hit Rate does not always indicate a good score; if combined with a high False Alarm Rate, it is likely a consequence of high levels of non-selective responding. Therefore, based on signal detection theory (Macmillan, 2004) Hit Rate and False Alarm Rate were used to calculate the discrimination sensitivity index, called D prime (d'), to provide an assessment of selective responding to the target stimulus. Sensitivity refers to the perceptual discriminability between the S+ and S- i.e., higher values indicate better visual discrimination. The rats had to achieve a D prime value of 0.8 to progress to the next stage. The discrimination sensitivity index was calculated as:

D prime (d') = (NORMSINV Hit Rate) - (NORMSINV False Alarm Rate)

Additional measures included, response criterion, which was calculated from the Hit Rate and False Alarm Rate. This measure refers to the criterion or willingness to make responses, e.g., conservative (high c values) or liberal (low c values) strategies. For instance, larger values indicate decreased overall responding to both the target and nontarget stimuli. The response criterion was calculated as: c = [(NORMSINV Hit Rate) + (NORMSINV False Alarm Rate)]/2

If a rat made responses to the screen during the ISI i.e., when no stimulus was present, these were recorded as ISI touches. This measure provides an indication of the rodent’s impulsivity during the test (i.e., inhibitory control).

In the final stage of training, the S- stimulus was changed. Instead of just one S- stimulus, there were four S- stimuli (**Figure 1B**). For the S+ stimulus, half of the rats were presented with vertical stripes and the other half were presented with horizontal stripes. All the parameters remained unchanged compared to the previous stage except the SD which was decreased to 1 s. Rats required 4–6 weeks to reach a stable baseline performance with minimum D prime value of 0.8. The baseline performance of each rat needed to be stable for at least two consecutive days before entering the experiment.

**Figure 1 Continuous Performance Test**

**A. B.**


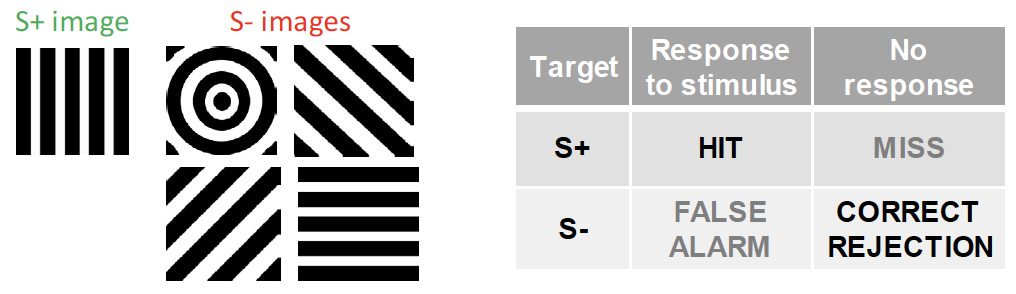


**(A).** Subjects were required to monitor and attend to briefly presented target stimuli (S+) amongst a stream of nontarget stimuli (S-) on the touchscreen. Half of the rats had vertical stripes as the S+ (as shown) and the other half had horizontal stripes. There were four S- stimuli. (**B).** There were four different possible outcomes: a correct response i.e., hit (nose poke to the S+ stimulus), an incorrect response i.e., false alarm (nose poke to the S- stimulus), a missed trial (no response to the S+ stimulus) and a correct rejection (no response to the S- stimulus).

Paired associates learning task

In the paired associates learning (PAL) task, twelve male LH rats (~400g, Charles River, Germany) were trained to learn which location is associated with each stimulus. Rats were tested in an operant chamber equipped with a touchscreen on one side and a pellet receptacle containing a light and an infra-red nose-poke detector on the opposite side. A black Perspex mask with three windows (6.1 x 10 cm) was positioned in front of the touchscreen giving three response areas. Two stimuli were presented at once in three possible locations, with one stimulus in the correct location (S+) and the other stimulus in an incorrect location (S-) (**Figure 2A**). There were six possible stimulus-location options (**Figure 2B**). The animal was required to select the correct stimulus-location to receive a food reward. Three images in total were used: plane, spider, flower. The rats received a maximum of 72 trials per session. A nose-poke to the blank screen had no effect on the trial. The image-location combination differed between animals in the cohort to reduce image bias.

**Figure 2 Paired associates learning task in the touchscreen**

1.
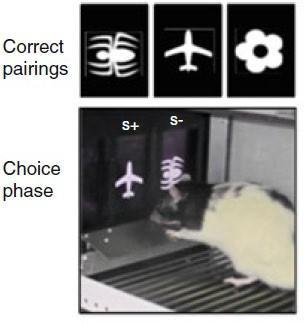

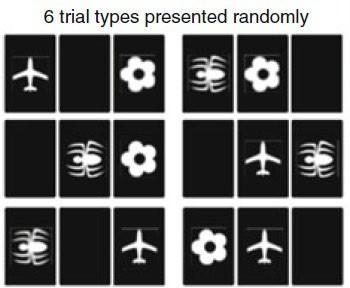
**B.**

At the start of the PAL task rats were required to initiate the test by nose-poking the pellet receptacle, which resulted in the images being shown. If rats made a correct response, they received a pellet and then an ITI of 10 s was initiated, where the house lights were switched on and the screen was blank. After the ITI, the rats had to initiate the next trial. The location of the two images was randomized. If the rats made an incorrect response, i.e., they pressed on the S- image, they received a 5 s time-out, where the screen went blank and the house light turned off, and then the ITI of 10 s was started. After the rats initiated the next trial, the same images were shown, in the same location as the previous trial; this trial is known as a correction trial. The correction trial was shown each time until the rat made the correct response. This helped the rats to learn the task faster. The correction trials however were not counted in the final dataset.

After rats had reached a pre-determined criterion of 80% correct then the correction trials were removed, so that if they made an incorrect response there was no correction trial and the next trial was chosen at random. Rats were trained until they had a stable baseline performance of 80% correct, which was achieved in approximately 60-80 days.

## ANXIETY TESTING

Social Approach Avoidance Test in rats

Thirty-seven male Lewis rats (200-220 g, Charles River, Germany) and 9 stimulus rats (male Sprague Dawley, approx. 500 g, Iffa Credo, France) were used in the social approach avoidance (SAA) test (Nicolas & Prinssen, 2006). The test box was divided into two separate compartments, social and non-social, connected by a sliding door. The social compartment contained a sub-chamber delimited by a perforated transparent wall, in which the stimulus rat (Sprague Dawley) was confined. Using a tracking system (Ethovision® videotracking system, Noldus; Netherlands), each of the compartments was subdivided into two virtual zones, i.e., proximal/distal for the social compartment and protected/hidden for the non-social compartment. The tracking system was used to measure the time spent in the different zones, as well as the speed and distance traveled during the habituation period. The arena was lit with dim white ceiling light (30-35 lux). The test started by introducing a Lewis rat into the non-social compartment for a 3 min habituation period. Note that the untreated stimulus rat was introduced into its sub chamber just before. After 3 minutes, the sliding door was opened, allowing the rat to move freely between the two compartments for 10 min. The time spent in each compartment as well as in different virtual zones of both compartments was measured. One factor ANOVA was used for between-group comparison (i.e., dose) and Dunnett's test was used for post hoc comparisons.

Elevated Plus Maze Test in rats

Forty male Sprague Dawley rats (220-250 g, Iffa Credo, France) were used in the elevated plus maze (EPM; (Martin, Ballard, & Higgins, 2002). The EPM consisted of two open arms perpendicular to two closed arms (each arm was 10 cm wide x 50 cm long) extending from a central platform. The apparatus was constructed from grey polyvinylchloride plastic and placed 50 cm above the floor. The closed arms, opposite to one other, had a surrounding wall of height 48 cm. The apparatus was situated in a sound-attenuated observation room with controlled illumination of 200 lux. The plus-maze was positioned in the middle of a closed black walled environment with the animal observed via a closed-circuit video camera mounted vertically over the maze. Behavioral analysis was conducted using a computerized system (Ethovision, Noldus Information Technology, The Netherlands). The test started by placing the Sprague Dawley rat onto the central platform facing a closed arm. The duration of the test was 5 min. The measures selected to represent anxiolytic-like behavior were the total time (s) spent in the open arms and percent entries into the open arms. The measure used to quantify motor activity was speed within the closed arms. One factor ANOVA was used for between-group comparison (i.e., dose) and Dunnett's test was used for post hoc comparisons.

Vogel Conflict Test in rats

Forty male Sprague Dawley rats (166-218 g, Iffa Credo, France) were used in the Vogel conflict drinking test (Goeldner, Spooren, Wichmann, & Prinssen, 2012). The operant conditioning boxes were obtained from Med Associates Inc. (VT, USA) and the protocols were run by the K-Limbic Control System from Conclusive Marketing Ltd. (Harlow, UK). Sprague Dawley rats were water-restricted for three consecutive 24 h periods. At the end of the second 24 h period, they were placed into the test chamber containing a drinking bottle and were allowed to drink freely for 15 min. The drinking time (defined as time spent by the rat at the drinking spout as measured by an optical sensor) in the test chamber was used to randomize the animals over the different treatments. At the end of the third 24 h period, rats were administered with alogabat or vehicle and isolated in a holding chamber until testing. During the 10 min test, rats were allowed to drink freely for a cumulative time of 5 s, after which drinking was punished. That is, an electrical shock between the grid floor and the drinking spout (0.5 mA, 250 ms) was triggered every second of cumulative drinking time. This shock level strongly suppresses drinking to about 10% of normal levels. Vogel conflict test data were analyzed by non-parametric Mann Whitney one-tailed tests.

## IN VIVO ELECTROPHYSIOLOGY STUDY: SPIKE-AND-WAVE DISCHARGE (SWD) ACTIVITY

Animals

In vivo electrophysiological recordings were performed in eight adult Sprague-Dawley (SD) rats (adult males; weight around 550-650 g at the time of recording) bred by Charles River, Germany. Rats were kept in a 12 h light/dark cycle at room temperature. Food and water were provided ad libitum. All procedures were approved by the Federal Food Safety and Veterinary Office of Switzerland and conducted in strict adherence to the Swiss federal ordinance on animal protection and welfare, as well as according to the rules of the Association for Assessment and Accreditation of Laboratory Animal Care International.

Surgery

Rats were anesthetized with 4% isoflurane for 5 minutes in an incubation chamber and received an injection of buprenorphine (0.2 mg/kg s.c.) for further analgesic treatment. Throughout the surgery, isoflurane levels were kept at 2-3% using an inhalation mask. For electrocorticography, stainless steel screws were placed above the frontal and parietal cortices. Coordinates (anterior-posterior, AP; medio-lateral, ML; dorso-ventral, DV) for left frontal cortex: AP=2.5 mm, ML=-1.2, left and right parietal cortex: AP=-4.0 mm, ML=±3.0 mm. Two additional screws were placed as reference and ground above the cerebellum at AP=-10, ML=±2. The implant was fixed to the skull with bone cement (Refobacin® Bone Cement R, Zimmer Biomet, Warsaw, Indiana, USA) and secured with ultraviolet-curing resin. For perioperative analgesia 0.1 mg/kg buprenorphine and 1 mg/kg meloxicam were injected s.c. directly after the end of the surgery. Postoperative analgesia (1 mg/kg meloxicam s.c. once daily) was performed for two additional days to minimize post-surgical pain.

Electrophysiological recording and quantification of epileptiform events

Short-term electrophysiological recordings (S3A-C) were performed according to (Janz, Bainier, et al., 2022) using an OmniPlex system (Plexon Inc, Dallas, Texas, USA). For long-term recordings in the follow-up study (S3D-F) wireless Neurologger devices (TSE Systems, Berlin, Germany) were used. Raw electrophysiological data was preprocessed as described previously (Janz, Nicolas, Redondo, & Valencia, 2022). Test sessions consisted of a 60 min baseline recording, followed by application of either vehicle or alogabat (4.4 mg/kg, p.o.). After waiting for 30 min post-dosing to achieve sufficient exposure of the compound, rats were recorded continuously for 60 min. Of note, from the eight rats only one rat (#3) showed clearly identifiable periods of spike-and-wave discharges (SWDs). Therefore, while all rats received the vehicle treatment to establish a robust control, rat #3 also received alogabat in a separate test session. For the prolonged electrophysiological recordings three animals from a separate cohort of rats were used. The three rats were selected based on the presence of clear SWDs. Selected rats were treated with either vehicle or two doses of alogabat (3 and 10 mg/kg, p.o.) in a randomized fashion. Electrophysiological recordings were started immediately after dosing and continued for at least 10 hours.

For detection of epileptiform events, we adapted a freely available toolbox from (Heining et al., 2019) to detect and quantify epileptic activity. In brief, in a first pass the algorithm detected spikes with increased power in the 4-40 Hz frequency band in the spectrum (normalized by the artifact-free interval of the recording) in three channels of interest (frontal cortex, parietal cortex and VMS). The following parameters were used for initial spike detection: Polarity = negative, threshold = 9.0; minimum refractory period = 0.07, lowest slope = 35, spike margin = 0.1. For the detection of bursts, we leveraged spikes identified with a threshold set to 5.5. Spikes that were separated in time by maximal 0.5 s were assigned as a “burst”. Individual bursts that were separated by maximal 1 s were merged. Until this step, the detected bursts in individual channels (particularly for the frontal and parietal cortex) may contain both epileptiform (i.e. SWDs) but also some physiological activity, such as high amplitude sleep spindles. However, only periods of sustained SWDs but not spindles are expected to be synchronous across large areas in the brain. As a last step, all events that were overlapping in time across all channels of interest were therefore classified as SWDs.

Alogabat suppresses spike-and-wave discharges

From a cohort of eight SD rats, we noted that one rat (#3) displayed frequent episodes of abnormal activity. Qualitative inspection revealed that these episodes, lasting up to 6 s, consisted of recurrent high amplitude events with a SWD characteristic at a modal frequency of around 8 Hz (**Supplementary Figure 3A,B**). These events were highly synchronous across all electrodes (frontal cortex, parietal cortex and VMS) and were paralleled by sudden phases of inactiveness on the behavioral level. Quantitative analysis of both individual spikes as well as bursts (see Methods above) demonstrated that, compared to rats with apparently normal electrographic activity (hereupon referred to as “control”), rat #3 had around 6-fold increased spike rate (in the frontal cortex) and a 9-fold increased rate of synchronized bursts compared to controls (**Supplementary Figure 3C**). Of note, although our analysis was optimized to detect SWD activity, in control rats the algorithm also detected a few events that did not have SWD characteristics but may represent other forms of atypical physiological activity.

To test whether enhancement of α5-subunit containing GABA_A_ receptors could suppress the occurrence of such epileptic activity, we applied alogabat at a dose of 4.4 mg/kg p.o. in rat #3 showing SWDs. Alogabat substantially reduced both the spike rate (pre-dose: 576 events/h, post-dose: 292 events/h) and the burst rate (pre-dose: 116 events/h, post-dose: 19 events/h) to control levels (**Supplementary Figure 3C**). This suppression of SWD activity lasted for around 50 min, until SWDs started to emerge again, indicating that at this time exposures fell below a level sufficient to elicit anti-epileptic effects. In contrast, the same animal treated with vehicle showed a sustained spike rate between pre- and post-dosing sessions (pre-dose: 557 events/h, post-dose: 545 events/h), but a reduction in the burst rate (pre-dose: 126 events/h, post-dose: 73 events/h). Here, it is important to note that while the rate of bursts dropped, the average duration of those events increased from 1.9 to 2.8 s, showing that under vehicle the time spent in the epileptic state remained equal between the pre- and post-dosing phase. Interestingly, in control animals, there was no change in burst rate from pre- to post-dosing, whereas we observed an increase in average spike rate from 92 to 222 events/h. Analysis of the rats’ behavior using video tracking revealed that in the post-dosing session, the animals were less active and spent more time in sleep/rest (predose: total sleep/rest: 16 mins, postdose: total sleep/rest: 27 mins). Visual inspection of the electrophysiological recordings suggested that the increase in spike rate was driven largely by sleep spindles that were emitted during sleep/rest periods.

These initial findings motivated us to run a follow-up study with rats from another cohort that showed pronounced SWDs. Leveraging wireless recording devices, we followed the occurrence of SWDs over at least 10 hours in the home-cages of the animals. Rats showed increased rates of SWDs within the first three hours of electrophysiological recording under vehicle control conditions (**Supplementary Figure 3D,E**). Treatment with both 3 and 10 mg/kg of alogabat substantially reduced the number of SWDs within this period without dose-dependency (average number of SWDs in 3 hours; Veh: 186.7; 3 mg/kg: 75.67; 10 mg/kg: 75.00). This is suggestive of a floor effect. Bioanalytic measurements of plasma samples confirmed higher exposures with 10 mg/kg compared to 3 mg/kg (**Supplementary Figure** **3G**).

Our observation that a fraction of SD rats displayed pronounced SWD activity, is in line with previous studies showing that SWDs can be present in a subset of SD rats (Pearce et al., 2014; Taylor, Smith, & Barth, 2022). Importantly, we observed that SWDs were highly synchronized between all brain regions we recorded from and coincided with sudden behavioral arrest, leading us to conclude that they represent generalized epileptic activity during absence seizures also found in GAERS and WAG/Rji rat strains (Akman, Demiralp, Ates, & Onat, 2010).

## Supplementary References

Akman, O., Demiralp, T., Ates, N., & Onat, F. Y. (2010). Electroencephalographic differences between WAG/Rij and GAERS rat models of absence epilepsy. *Epilepsy Res, 89*(2-3), 185-193. doi:10.1016/j.eplepsyres.2009.12.005

Alsop, D. C., & Detre, J. A. (1996). Reduced transit-time sensitivity in noninvasive magnetic resonance imaging of human cerebral blood flow. *J Cereb Blood Flow Metab, 16*(6), 1236-1249. doi:10.1097/00004647-199611000-00019

Atack, J. R., Alder, L., Cook, S. M., Smith, A. J., & McKernan, R. M. (2005). In vivo labelling of alpha5 subunit-containing GABA(A) receptors using the selective radioligand [(3)H]L-655,708. *Neuropharmacology, 49*(2), 220-229. doi:10.1016/j.neuropharm.2005.03.004

Ballard, T. M., Knoflach, F., Prinssen, E., Borroni, E., Vivian, J. A., Basile, J., … Hernandez, M. C. (2009). RO4938581, a novel cognitive enhancer acting at GABAA alpha5 subunit-containing receptors. *Psychopharmacology (Berl), 202*(1-3), 207-223. doi:10.1007/s00213-008-1357-7

Bomatter, P., Paillard, J., Garces, P., Hipp, J., & Engemann, D. A. (2024). Machine learning of brain-specific biomarkers from EEG. *EBioMedicine, 106*, 105259. doi:10.1016/j.ebiom.2024.105259

Bruns, A., Kunnecke, B., Risterucci, C., Moreau, J. L., & von Kienlin, M. (2009). Validation of cerebral blood perfusion imaging as a modality for quantitative pharmacological MRI in rats. *Magn Reson Med, 61*(6), 1451-1458. doi:10.1002/mrm.21779

Deichmann, R., Hahn, D., & Haase, A. (1999). Fast T1 mapping on a whole-body scanner. *Magn Reson Med, 42*(1), 206-209. doi:10.1002/(sici)1522-2594(199907)42:1<206::aid-mrm28>3.0.co;2-q

Goeldner, C., Spooren, W., Wichmann, J., & Prinssen, E. P. (2012). Further characterization of the prototypical nociceptin/orphanin FQ peptide receptor agonist Ro 64-6198 in rodent models of conflict anxiety and despair. *Psychopharmacology (Berl), 222*(2), 203-214. doi:10.1007/s00213-012-2636-x

Haase, A., Frahm, J., Matthaei, D., Hanicke, W., & Merboldt, K. D. (2011). FLASH imaging: rapid NMR imaging using low flip-angle pulses. 1986. *J Magn Reson, 213*(2), 533-541. doi:10.1016/j.jmr.2011.09.021

Heining, K., Kilias, A., Janz, P., Haussler, U., Kumar, A., Haas, C. A., & Egert, U. (2019). Bursts with High and Low Load of Epileptiform Spikes Show Context-Dependent Correlations in Epileptic Mice. *eNeuro, 6*(5)doi:10.1523/ENEURO.0299-18.2019

Hennig, J., Nauerth, A., & Friedburg, H. (1986). RARE imaging: a fast imaging method for clinical MR. *Magn Reson Med, 3*(6), 823-833. doi:10.1002/mrm.1910030602

Higgins, G. A., Ballard, T. M., Enderlin, M., Haman, M., & Kemp, J. A. (2005). Evidence for improved performance in cognitive tasks following selective NR2B NMDA receptor antagonist pre-treatment in the rat. *Psychopharmacology (Berl), 179*(1), 85-98. doi:10.1007/s00213-005-2203-9

Hipp, J. F., Hawellek, D. J., Corbetta, M., Siegel, M., & Engel, A. K. (2012). Large-scale cortical correlation structure of spontaneous oscillatory activity. *Nat Neurosci, 15*(6), 884-890. doi:10.1038/nn.3101

Hipp, J. F., Knoflach, F., Comley, R., Ballard, T. M., Honer, M., Trube, G., … Hernandez, M. C. (2021). Basmisanil, a highly selective GABA(A)-alpha5 negative allosteric modulator: preclinical pharmacology and demonstration of functional target engagement in man. *Sci Rep, 11*(1), 7700. doi:10.1038/s41598-021-87307-7

Janz, P., Bainier, M., Marashli, S., Schoenenberger, P., Valencia, M., & Redondo, R. L. (2022). Neurexin1alpha knockout rats display oscillatory abnormalities and sensory processing deficits back-translating key endophenotypes of psychiatric disorders. *Transl Psychiatry, 12*(1), 455. doi:10.1038/s41398-022-02224-1

Janz, P., Nicolas, M. J., Redondo, R. L., & Valencia, M. (2022). GABA(B) R activation partially normalizes acute NMDAR hypofunction oscillatory abnormalities but fails to rescue sensory processing deficits. *J Neurochem, 161*(5), 417-434. doi:10.1111/jnc.15602

Macmillan, N. A., Creelman, C.D. (2004). *Detection theory: a user’s guide*. New York: Psychology Press.

Malherbe, P., Bissantz, C., Marcuz, A., Kratzeisen, C., Zenner, M. T., Wettstein, J. G., … Spooren, W. (2008). Me-talnetant and osanetant interact within overlapping but not identical binding pockets in the human tachykinin neurokinin 3 receptor transmembrane domains. *Mol Pharmacol, 73*(6), 1736-1750. doi:10.1124/mol.107.042754

Martin, J. R., Ballard, T. M., & Higgins, G. A. (2002). Influence of the 5-HT2C receptor antagonist, SB-242084, in tests of anxiety. *Pharmacol Biochem Behav, 71*(4), 615-625. doi:10.1016/s0091-3057(01)00713-4

Nicolas, L. B., & Prinssen, E. P. (2006). Social approach-avoidance behavior of a high-anxiety strain of rats: effects of benzodiazepine receptor ligands. *Psychopharmacology (Berl), 184*(1), 65-74. doi:10.1007/s00213-005-0233-y

Paxinos, G., & Watson, C. (1986). *The rat brain in stereotaxic coordinates.*: Academic Press.

Pearce, P. S., Friedman, D., Lafrancois, J. J., Iyengar, S. S., Fenton, A. A., Maclusky, N. J., & Scharfman, H. E. (2014). Spike-wave discharges in adult Sprague-Dawley rats and their implications for animal models of temporal lobe epilepsy. *Epilepsy Behav, 32*, 121-131. doi:10.1016/j.yebeh.2014.01.004

Taylor, J. A., Smith, Z. Z., & Barth, D. S. (2022). Spike-wave discharges in Sprague-Dawley rats reflect precise intra- and interhemispheric synchronization of somatosensory cortex. *J Neurophysiol, 128*(5), 1152-1167. doi:10.1152/jn.00303.2022

van Lier, H., Drinkenburg, W. H., van Eeten, Y. J., & Coenen, A. M. (2004). Effects of diazepam and zolpidem on EEG beta frequencies are behavior-specific in rats. *Neuropharmacology, 47*(2), 163-174. doi:10.1016/j.neuropharm.2004.03.017

Visser, S. A., Wolters, F. L., Gubbens-Stibbe, J. M., Tukker, E., Van Der Graaf, P. H., Peletier, L. A., & Danhof, M. (2003). Mechanism-based pharmacokinetic/pharmacodynamic modeling of the electroencephalogram effects of GABAA receptor modulators: in vitro-in vivo correlations. *J Pharmacol Exp Ther, 304*(1), 88-101. doi:10.1124/jpet.102.042341

Williams, D. S., Detre, J. A., Leigh, J. S., & Koretsky, A. P. (1992). Magnetic resonance imaging of perfusion using spin inversion of arterial water. *Proc Natl Acad Sci U S A, 89*(1), 212-216. doi:10.1073/pnas.89.1.212

# Supplementary Figures and Tables

## Supplementary Figures

**A. B.**

**Supplementary Figure 1.** **(A)** **Alogabat is a potent PAM at both γ2 and γ3 subunit-containing GABA_A_-α5 receptors.** Concentration-response curves of the effects of alogabat in Xenopus oocytes expressing human recombinant GABA_A_ receptors with indicated subunit composition. Ion currents were induced by 3 µM GABA (n=5 per concentration). Data in the graph are shown as mean (symbols) ± SEM (error bars). Error bars smaller than the symbol size are not shown. EC_50_ values derived from the fits were 12 nM for GABA_A_-α5β3γ2 receptors and 20 nM for GABA_A_-α5β3γ3 receptors. **(B)** Concentration-response curves of the effects of alogabat in Xenopus oocytes expressing different human recombinant GABA_A_ receptor subtypes. Ion currents were induced by GABA concentrations evoking approximately 10% of a maximal response (n = 3 per concentration). Data in the graphs are shown as mean (symbols) ± SEM (error bars). Error bars smaller than the symbol size are not shown.


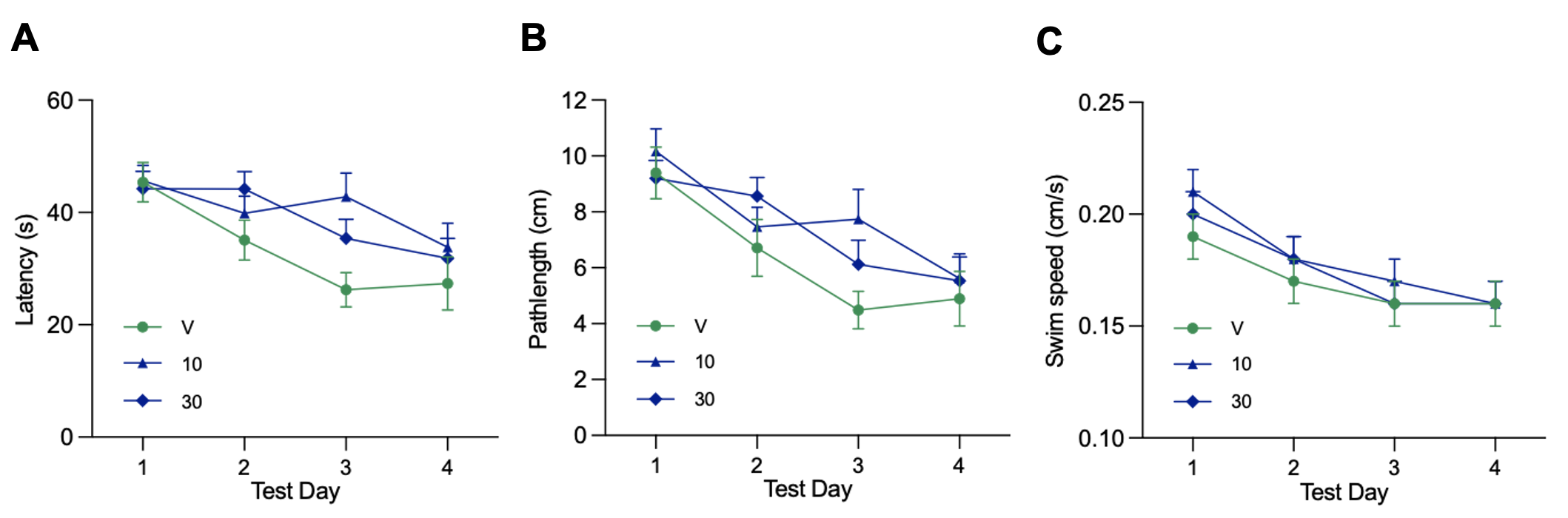


**Supplementary Figure 2.** **Alogabat does not impair acquisition of a hidden platform position in the Morris Water Maze.** Chronic administration of alogabat at 10 and 30 mg/kg p.o. did not have a significant effect on: (**A**). latency (F(6,81)=1.88, p>0.05); (**B**). pathlength (F(6,81)=1.25, p>0.1); and (**C**). swim speed (F(6,81)=0.52, p>0.1) traveled to find the hidden platform position (place acquisition). Data are expressed as mean ± SEM; n=10 rats/dose group.


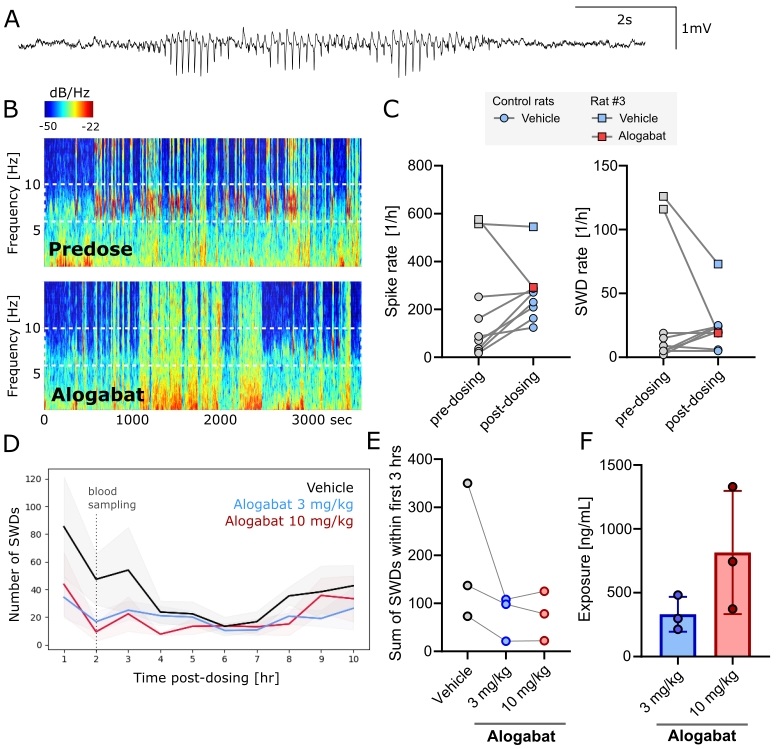


**Supplementary Figure 3. Alogabat suppresses epileptic activity in Sprague Dawley rats with prominent spike-and-wave discharge activity.** (**A**). Spike-and-wave discharges (SWDs) recorded in the frontal cortex from Sprague Dawley (SD) rat #3, 3 bursts, total burst duration approximately 6 s. (**B**). Spectrograms showing SWD discharges with highest power around 8 Hz. Before dosing, 1-2 SWDs per minute were emitted, whereas after dosing with alogabat SWDs were suppressed almost completely for around 50 mins. Dashed boxes highlight 6-10 Hz range. (**C**). Quantification of spike rate (for frontal cortex) and burst rate (synchronous events across electrodes): Blue: Recordings with vehicle administration, red: alogabat (4.4 mg/kg p.o.). Square symbols: SD rat #3 with frequent SWD activity, circles: 7 SD rats from the same experimental cohort that did not show clear SWD activity. (**D**). Quantification of SWD occurrence in 3 rats with pronounced SWDs over 10 hours of continuous EEG recording with two different doses (3 and 10 mg/kg). Dashed line indicates the time point of blood sampling to retrieve exposures shown in F). (**E**). Sum of SWDs within the first 3 h post dosing for individual rats in all tested conditions. (**F**). Quantification of plasma exposures measured at 2 h post-dosing. For further information on this study refer to Supplementary Information above.


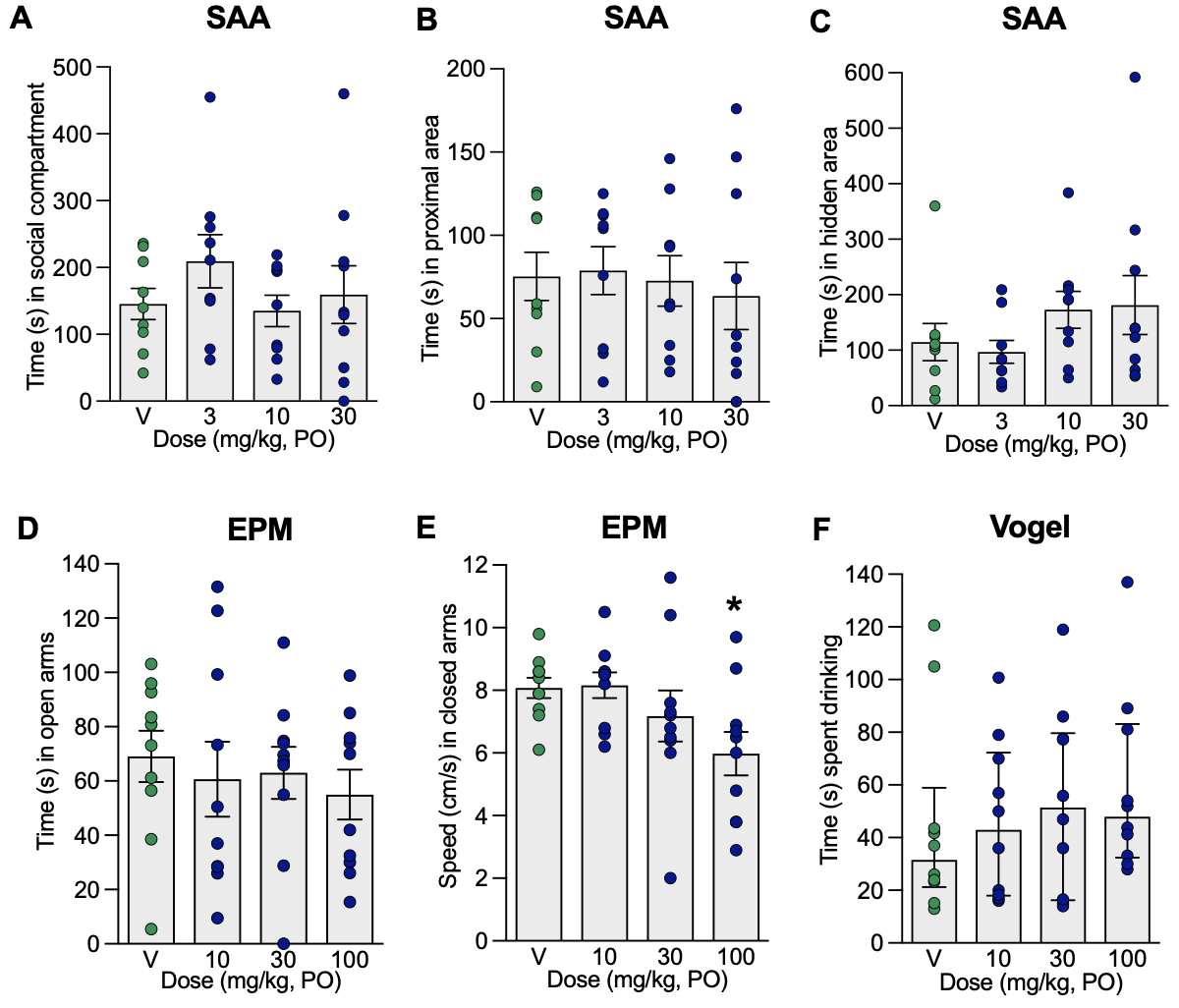


**Supplementary Figure 4.** **Alogabat does not have an anxiolytic-like profile in rats at plasma concentrations up to 80% RO.** Alogabat was administered at 3, 10 and 30 mg/kg p.o. 1 h prior to the social approach avoidance (SAA) test in rats. Time spent in: (**A**). social compartment; (**B**). proximal area; (**C**). hidden area. Data are presented as individual points (circles) and mean ± SEM (n=9-10/ group). Alogabat was administered at 10, 30 and 100 mg/kg p.o. 1 h prior to the elevated plus maze (EPM) test in rats: (**D**). time spent in the open arms; (**E**). speed in the closed arms. Data are presented as individual points (circles) and mean ± SEM (n=10/group). Statistics: p<0.05 vs. vehicle (V). (**F**). Alogabat was administered at 10, 30 and 100 mg/kg p.o. 1 h prior to the Vogel test in rats. Data are presented as individual points (circles) and median ± IQR (n=10/group). IQR: interquartile range; RO: receptor occupancy; SEM: standard error of the mean.

**Supplementary Figure 5.** **A GABA_A_-α5 negative allosteric modulator does not interfere with the anxiolytic effect of chlordiazepoxide.** GABA_A_-α5 negative allosteric modulator (NAM), basmisanil (Hipp et al 2021), was administered at 1, 3 and 10 mg/kg p.o. (1 h prior to testing) in combination with chlordiazepoxide (CDZ; 10 mg/kg p.o.; 30 min prior to testing) in the social approach avoidance (SAA) test in rats. (**A**). CDZ significantly increased the time spent in the social compartment. Basmisanil administered in combination with CDZ did not have a significant effect on the time spent in the social compartment (F(3,35)=0.7, p=0.5). (**B**). CDZ significantly reduced the time spent in the hidden area. Basmisanil administered in combination with CDZ did not have a significant effect on the time spent in the hidden area (F(3,35)=0.7, p=0.6) as compared to CDZ alone. Data are presented as individual points (circles) and as mean ± SEM (n=9-10/ group). Statistics: * p<0.05, *** p<0.001: CDZ vs. vehicle (V). SEM: standard error of the mean.

## Supplementary Tables

**Supplementary Table 1.** Affinity of alogabat for rat and human recombinant GABA_A_ receptor subtypes.

| **GABA_A_**  **receptor subtype** | **Rat** | | | **Human** | | |
| --- | --- | --- | --- | --- | --- | --- |
|  | **Ki (nM ± SEM)** | **N** | **α5 selectivity** | **Ki (nM ± SEM)** | **N** | **α5 selectivity** |
| **α5β3γ2** | **7.9 ± 1.1** | **12** | **-** | **10.9 ± 1** | **20** | **-** |
| α1β3γ2 | 292.5 ± 16.2 | 11 | 37-fold | 415.8 ± 38.7 | 19 | 38-fold |
| α2β3γ2 | 208.1 ± 41.9 | 11 | 26-fold | 178.3 ± 7.1 | 21 | 16-fold |
| α3β3γ2 | 142.3 ± 10.25 | 11 | 18-fold | 113 ± 10.4 | 17 | 10-fold |
| α4β2γ2 | ND | ND | - | 538 ± 31.9 | 12 | 49-fold |
| α6β2γ2 | ND | ND | - | 6935 ± 772 | 12 | 637-fold |

Inhibition of [^3^H]flumazenil binding was used to measure affinity of alogabat at GABA_A_ α1, α2, α3, and α5 subunit-containing receptor subtypes. Inhibition of [^3^H]RO0154513 binding was used to measure affinity of alogabat at GABA_A_ α4, and α6 subunit-containing receptor subtypes. ND=not determined.

**Supplementary Table 2.** Functional selectivity for the rat and human GABA_A_-α5 receptor subtype.

| **GABA_A_**  **receptor subtype** | **Rat** | | | | **Human** | | | |
| --- | --- | --- | --- | --- | --- | --- | --- | --- |
|  | **Max % modulation** | **EC_50_ (nM)** | **Hill** | **N** | **Max % modulation** | **EC_50_ (nM)** | **Hill** | **N** |
| **α5β3γ2** | **167** | **25** | **1.0** | **4** | **72** | **32** | **1.1** | **3** |
| α1β3γ2 | 55 | 288 | 0.7 | 5 | 35 | 384 | 1.0 | 3 |
| α2β3γ2 | 103 | 503 | 1.1 | 4 | 36 | 142 | 0.7 | 3 |
| α3β3γ2 | 75 | 1047 | 1.6 | 4 | 43 | 79 | 0.7 | 3 |

Modulation of GABA_A_-induced current was used to measure the intrinsic activity of alogabat at GABA_A_ α1, α2, α3, and α5 subunit-containing receptor subtypes expressed in HEK293 cells (rat receptors) or Xenopus oocytes (human receptors).

**Supplementary Table 3. Selectivity profile of alogabat.**

Source: species and tissue or cell line or recombinant receptors (cell line of expression in parentheses). % Inhibition: percentage inhibition of specific binding of the radioligand (or other ligand) in presence of 10 μM basmisanil. Mean values of n = 2. All experiments were done by Eurofins Cerep SA, Celle l’Evescault, France. For more information see the website, [www.neurofinsdiscoveryservices.com](http://www.neurofinsdiscoveryservices.com).

**Binding Assays**

| **Assay** | **Source** | **Reference Compound** | **Ligand** | **% Inhibition** |
| --- | --- | --- | --- | --- |
| **Receptors** |  |  |  |  |
| **A_1_*(h)***  **(agonist radioligand)** | human recombinant (CHO cells) | CPA | [^3^H]CCPA | 5.3 |
| **A_3_*(h)***  **(agonist radioligand)** | human recombinant (HEK-293 cells) | IB-MECA | [^125^I]AB-MECA | -7.5 |
| **α_1A_*(h)***  **(antagonist radioligand)** | human recombinant (CHO cells) | WB 4101 | [^3^H]prazosin | -2.7 |
| **α_2A_ *(h)***  **(antagonist radioligand)** | human recombinant (CHO cells) | yohimbine | [^3^H]RX 821002 | -0.1 |
| **β_1_ *(h)***  **(agonist radioligand)** | human recombinant (HEK-293 cells) | atenolol | [^3^H](-)CGP 12177 | -6.2 |
| **AT_1_ *(h)***  **(antagonist radioligand)** | human recombinant (HEK-293 cells) | saralasin | [^125^I][Sar^1^,Ile^8^]-AT-II | -11.6 |
| **D_1_*(h)***  **(antagonist radioligand)** | human recombinant (CHO cells) | SCH 23390 | [^3^H]SCH 23390 | 2.3 |
| **D_2S_*(h)***  **(agonist radioligand)** | human recombinant (HEK-293 cells) | 7-OH-DPAT | [^3^H]7-OH-DPAT | -4.8 |
| **H_1_*(h)***  **(antagonist radioligand)** | human recombinant (HEK-293 cells) | pyrilamine | [^3^H]pyrilamine | -0.1 |
| **H_2_*(h)***  **(antagonist radioligand)** | human recombinant (CHO cells) | cimetidine | [^125^I]APT | 14.5 |
| **H_3_*(h)***  **(agonist radioligand)** | human recombinant (CHO cells) | (R)alpha-Me-histamine | [^3^H]N^α^-Me-histamine | -21.6 |
| **I_1_**  **(agonist radioligand)** | bovine adrenal medulla glands | rilmenidine | [^3^H]clonidine | -22.2 |
| **MT_3_ (ML_2_)**  **(agonist radioligand)** | hamster brain | melatonin | [^125^I]2-iodomelatonin | 46.3 |
| **M_2_ *(h)***  **(antagonist radioligand)** | human recombinant (CHO cells) | methoctramine | [^3^H]AF-DX 384 | 2.4 |
| **M_4_*(h)***  **(antagonist radioligand)** | human recombinant (CHO cells) | 4-DAMP | [^3^H]4-DAMP | 9.1 |
| **N muscle-type *(h)***  **(antagonist radioligand)** | TE671 cells (endogenous) | alpha-bungarotoxin | [^125^I]α-bungarotoxin | -1.8 |
| **κ (KOP)**  **(agonist radioligand)** | rat recombinant (CHO cells) | U 50488 | [^3^H]U 69593 | 19.2 |
| **μ (MOP) *(h)***  **(agonist radioligand)** | human recombinant (HEK-293 cells) | DAMGO | [^3^H]DAMGO | 10.8 |
| **PPARγ *(h)***  **(agonist radioligand)** | human recombinant (*E. coli*) | rosiglitazone | [^3^H]rosiglitazone | -13.8 |
| **FP *(h)***  **(agonist radioligand)** | human recombinant (HEK-293 cells) | PGF2alpha | [^3^H]PGF_2α_ | 12.0 |

**Binding Assays (continued)**

| **Assay** | **Source** | **Reference Compound** | **Ligand** | **% Inhibition** |
| --- | --- | --- | --- | --- |
| **Receptors** |  |  |  |  |
| **5-HT_1A_ *(h)***  **(agonist radioligand)** | human recombinant (HEK-293 cells) | 8-OH-DPAT | [^3^H]8-OH-DPAT | -13.4 |
| **5-HT_2A_ *(h)***  **(agonist radioligand)** | human recombinant (HEK-293 cells) | (±)DOI | [^125^I](±)DOI | -20.4 |
| **5-HT_2B_ *(h)***  **(agonist radioligand)** | human recombinant (CHO cells) | (±)DOI | [^125^I](±)DOI | -13.3 |
| **sigma (non-selective) (h) (agonist radioligand)** | Jurkat cells (endogenous) | haloperidol | [3H]DTG | 15.8 |
| **sst_4_ *(h)***  **(agonist radioligand)** | human recombinant (CHO cells) | somatostatin-14 | [^125^I]Tyr^11^-somatostatin-14 | 4.6 |
| **GR *(h)***  **(agonist radioligand)** | IM-9 cells (cytosol) | dexamethasone | [^3^H]dexamethasone | 0.3 |
| **ERα *(h)***  **(agonist fluoligand)** | human recombinant (Sf9 cells) | 17-beta -estradiol | fluormone^TM^ES2 | 0.7 |
| **PXR *(h)***  **(agonist radioligand)** | human recombinant (insect cells) | T0901317 | [^3^H]SR 12813 | -10.0 |
| **AR *(h)***  **(agonist radioligand)** | LNCaP cells (cytosol) | mibolerone | [^3^H]methyltrienolone | 3.0 |
| **Ion channels** |  |  |  |  |
| **BZD (central) (agonist radioligand)** | rat cerebral cortex | diazepam | [^3^H]flunitrazepam | **93.7** |
| **glycine**  **(strychnine-insensitive) (antagonist radioligand)** | rat cerebral cortex | glycine | [^3^H]MDL 105,519 | -4.4 |
| **PCP**  **(antagonist radioligand)** | rat cerebral cortex | MK 801 | [^3^H]TCP | -6.4 |
| **5-HT_3_*(h)***  **(antagonist radioligand)** | human recombinant (CHO cells) | MDL 72222 | [^3^H]BRL 43694 | -10.0 |
| **Ca^2+^ channel**  **(L, diltiazem site) (benzothiazepines) (antagonist radioligand)** | rat cerebral cortex | diltiazem | [^3^H]diltiazem | -9.1 |
| **Na^+^ channel (site 2) (antagonist radioligand)** | rat cerebral cortex | veratridine | [^3^H]batrachotoxinin | -2.0 |
| **Transporters** |  |  |  |  |
| **norepinephrine transporter *(h)* (antagonist radioligand)** | human recombinant (CHO cells) | protriptyline | [^3^H]nisoxetine | -3.8 |
| **5-HT transporter *(h)***  **(antagonist radioligand)** | human recombinant (CHO cells) | imipramine | [^3^H]imipramine | 2.6 |

**Cellular and Nuclear Receptor Functional Assays**

| **Assay** | **Source** | **Reference Compound** | **Ligand** | **% of control response** |
| --- | --- | --- | --- | --- |
| **Receptors** |  |  |  |  |
| **CAR *(h)***  **(agonist effect)** | human recombinant | CITCO | None (100 µM CITCO for control) | -0.7 |
| **CAR *(h)***  **(antagonist effect)** | human recombinant | PK 11195 | CITCO (3 µM) | 4.9 |
| **PPARα *(h)***  **(agonist effect)** | human recombinant | GW 7647 | None (1 µM GW 7647 for control) | -0.2 |
| **PPARα *(h)***  **(antagonist effect)** | human recombinant | GW 9662 | GW 7647 (100 nM) | -33.2 |
| **PXR *(h)***  **(agonist effect)** | human recombinant | T0901317 | None (1 µM T0901317 for control) | -5.3 |
| **PXR *(h)***  **(antagonist effect)** | human recombinant | clotrimazole | T0901317 (300 nM) | 10.5 |

**Enzyme and Uptake Assays**

| **Assay** | **Source** | **Reference Compound** | **Ligand** | **% Inhibition** |
| --- | --- | --- | --- | --- |
| **Kinases** |  |  |  |  |
| **CDK2 *(h)***  **(cycA)** | human recombinant | staurosporine | ATP + Ulight- CFFKNIVTPRTPPPSQGK-amide | -4.2 |
| **GSK3α *(h)*** | human recombinant | staurosporine | ATP + Ulight- CFFKNIVTPRTPPPSQGK-amide | -4.7 |
| **GSK3β *(h)*** | human recombinant | staurosporine | ATP + Ulight- CFFKNIVTPRTPPPSQGK-amide | -1.1 |
| **ZAP70 kinase *(h)*** | human recombinant (insect cells) | staurosporine | ATP + biotinyl- βAβAβADEEEYFIPP | 3.9 |
| **Other enzymes** |  |  |  |  |
| **COX_2_ *(h)*** | human recombinant (Sf9 cells) | NS 398 | arachidonic acid (2 µM) | 22.4 |
| **PDE5 *(h)***  **(non-selective)** | human platelets | dipyridamole | [^3^H]cGMP  + cGMP (1 µM) | -12.5 |
| **ACE *(h)*** | human recombinant | captopril | Abz-FRK(Dnp)-P-OH (15 µM) | -26.5 |
| **HIV-1 protease** | protein viral recombinant (E.coli) | pepstatin A | antranilyl-HIV (75 µM) | -1.2 |
| **acetylcholinesterase *(h)*** | human recombinant (HEK-293 cells) | neostigmine | AMTCh (400 µM) | -2.1 |
| **MAO-A *(h)*** | human placenta | clorgyline | kynuramine (0.15 mM) | 15.4 |
| **MAO-B *(h)* recombinant enzyme** | human recombinant | deprenyl | D-Luciferin derivative (4 µM) | -25.3 |
| **xanthine oxidase /superoxide O_2_^–^ scavenging** | purified xanthine oxidase from bovine milk | allopurinol | hypoxanthine | 5.8 |

**Supplementary Table 4.** Alogabat plasma concentrations and calculated percent receptor occupancy in mice.

| **Dose (mg/kg)** | **BTBR Grooming**  (30 min post admin; **acute**) | | **BTBR Digging**  (40 min post admin; **acute**) | | **Cntnap 2 Grooming**  (30 min post admin; **subchronic**) | |
| --- | --- | --- | --- | --- | --- | --- |
|  | **Plasma**  **(ng/mL)** | **RO**  **(%)** | **Plasma**  **(ng/mL)** | **RO**  **(%)** | **Plasma**  **(ng/mL)** | **RO**  **(%)** |
| 30 | 657 (44%) | 28 | 680 (14%) | 28 | 1480 (47%) | 46 |
| 60 | 1440 (22%) | 45 | 2510 (67%) | 58 | 1940 (22%) | 52 |
| 90 | 2930 (30%) | 62 | - | - | - | - |
| 100 | - | - | 3790 (58%) | 67 | 7830 (110%) | 79 |
| 120 | 4540 (36%) | 70 | - | - | - | - |

Data are expressed as mean with coefficient of variation in parentheses. BTBR grooming: n=6/dose group; BTBR digging: n=3/dose group; Cntnap2 -/- grooming: n=4/dose group. RO: receptor occupancy.

**Supplementary Table 5.** Alogabat plasma concentrations and calculated percent receptor occupancy in rats.

| **Dose (mg/kg)** | **PTZ**  (60 min post admin; **acute**) | | **MES**  (60 min post admin; **acute**) | | **Rotarod**  (60 min post admin; **acute**  **co-admin DZP**) | |
| --- | --- | --- | --- | --- | --- | --- |
|  | **Plasma**  **(ng/mL)** | **RO**  **(%)** | **Plasma**  **(ng/mL)** | **RO**  **(%)** | **Plasma**  **(ng/mL)** | **RO**  **(%)** |
| 3 | - | - | - | - | 524 (20%) | 41 |
| 5 | 1368 (27%) | 57 | 1358 (17%) | 57 | - |  |
| 10 | - | - | - | - | 1490 (20%) | 66 |
| 15 | 3619 (24%) | 71 | 4891 (24%) | 75 | - | - |
| 30 | 6265 (33%) | 77 | 8593 (12%) | 80 | 5200 (23%) | 87^a^ |

Data are expressed as mean with coefficient of variation in parentheses. PTZ: n=8/dose group; MES: n=8/dose group; Rotarod: n=8/dose group. DZP: diazepam; RO: receptor occupancy.

^a^ RO is mean of DZP + 30 mg/kg group (86%) and 30 mg/kg alone group (88%).

**Supplementary Table 6.** Summary of alogabat in vivo pharmacology, active dose, CSF concentration, calculated percent receptor occupancy for GABA_A_ α5 and α1

CFC = contextual fear conditioning; CSF = cerebrospinal fluid; MWM = Morris water maze; PTZ = pentylenetetrazole; RO = receptor occupancy.

CSF was calculated from total plasma concentration using experimental CSF to plasma ratio determined in pharmacokinetic experiments in the corresponding species.

RO was calculated from total plasma concentration using the following equation:

RO(%) = ([D] ^γ^ * Emax)/((EC_50_) ^γ^ + [D] ^γ^).

[D] is the total plasma concentration of the drug. Emax is maximal occupancy, γ is hill coefficient and EC_50_ is half maximal concentration, from the corresponding in vivo receptor occupancy study with [^3^H]RO0154513.
